# Genome-wide identification and comparative analysis of *CLE* family in rapeseed and its diploid progenitors

**DOI:** 10.3389/fpls.2022.998082

**Published:** 2022-10-20

**Authors:** Meili Xie, Chuanji Zhao, Min Song, Yang Xiang, Chaobo Tong

**Affiliations:** ^1^ Guizhou Rapeseed Institute, Guizhou Academy of Agricultural Sciences, Guiyang, China; ^2^ The Key Laboratory of Biology and Genetic Improvement of Oil Crops, The Ministry of Agriculture and Rural Affairs, Oil Crops Research Institute, Chinese Academy of Agricultural Sciences, Wuhan, China; ^3^ College of Life Science, Qufu Normal University, Qufu, China

**Keywords:** CLE peptide, *Brassica napus*, diploid progenitor, orthologous pairs, expression pattern, association mapping analysis, yield traits

## Abstract

Crop genomics and breeding CLAVATA3/EMBRYO SURROUNDING REGION-RELATED (CLE) proteins belong to a small peptide family in plants. During plant development, *CLE* gene family members play a pivotal role in regulating cell-to-cell communication and stem cell maintenance. However, the evolutionary process and functional importance of *CLE*s are unclear in Brassicaceae. In this study, a total of 70 *BnCLE*s were identified in *Brassica napus* (2n = 4x = 38, A_n_C_n_): 32 from the A_n_ subgenome, 36 from the C_n_ subgenome, and 2 from the unanchored subgenome. Meanwhile, 29 *BrCLE* and 32 *BoCLE* genes were explored in *Brassica rapa* (2n = 2x = 20, A_r_) and *Brassica oleracea* (2n = 2x = 18, C_o_). Phylogenetic analysis revealed that 163 *CLE*s derived from three *Brassica* species and *Arabidopsis thaliana* can be divided into seven subfamilies. Homology and synteny analyses indicated whole-genome triplication (WGT) and segmental duplication may be the major contributors to the expansion of *CLE* family. In addition, RNA-seq and qPCR analysis indicated that 19 and 16 *BnCLE*s were more highly expressed in immature seeds and roots than in other tissues. Some *CLE* gene pairs exhibited different expression patterns in the same tissue, which indicated possible functional divergence. Furthermore, genetic variations and regional association mapping analysis indicated that 12 *BnCLE*s were potential genes for regulating important agronomic traits. This study provided valuable information to understand the molecular evolution and biological function of *CLE*s in *B. napus* and its diploid progenitors, which will be helpful for genetic improvement of high-yield breeding in *B. napus*.

## Introduction

Peptide hormones are active molecules composed of many to several tens of amino acids and serve as signal molecules to exchange information between cells (Grienenberger and Fletcher, 2015). In plants, there are various polypeptides; CLAVATA3/EMBRYO SURROUNDING REGION-RELATED (CLE), one of the largest families of known polypeptides, is generally 12–13 amino acids in length, including a secretion signal peptide in N-terminus and a highly conserved CLE domain in C-terminus. Domain deletion and domain exchange experiments have indicated that the CLE domain of *CLV3* in *Arabidopsis thaliana* plays an independent role in adjacent flanking sequences (Fletcher et al., 1999; Rojo et al., 2002). With the help of matrix-assisted laser desorption ionization–time-of-flight (MALDI-TOF) mass spectrometry, an active 12-amino acid CLV3 polypeptide molecule (corresponding to the CLE domain, with one amino acid removed from each side), is isolated from *A. thaliana* over-expressing *CLV3*, and the peptide synthesized *in vitro* is functional (Tatsuhiko, 2006). Exogenous application of synthetic CLV3, CLE19, and CLE40 polypeptides exhibits a similar phenotype with overexpression of these *CLE* genes. CLV3 polypeptides also restore the *clv3-2* mutant phenotype (Fiers et al., 2006). These results indicate that CLE polypeptides are the active form of CLE family proteins.

It is difficult to clearly identify the function of each CLE peptide owing to its small size and high sequence conservation. However, the application of new technologies such as gene editing makes it easier to study their functions. However, it is easier to study their functions based on the application of new technology, like gene editing. Now, many results show that CLE polypeptides play important roles in plant development and hormone and stress response. In shoot apical meristems (SAMs), the stem cell homeostasis is maintained by a dynamic negative feedback loop involved in the CLV3-WUSCHEL (WUS) pathway. The transcription factor gene *WUS*, which interacts with SHOOT MERISTEMLESS (STM), can promote the expression of CLV3; meanwhile, overmuch CLV3 will suppress the expression of STM and WUS to maintain the stem cell population in the SAM (Schoof et al., 2000). In addition to participating in the division and differentiation of meristem cells, CLE polypeptides also play a key role in the development of seeds (Fiume, 2010). With the use of the method of promoter fusion GUS, *CLE8* and *WUSCHEL-related homeobox 8* (*WOX8*) are expressed in the embryo and endosperm during the early stages of seed development in *Arabidopsis* (Fiume and Fletcher, 2012). The number of embryo and endosperm cells decreases in the *cle8-1* mutant, suggesting that *CLE8* promotes the proliferation of embryo and endosperm cells. The length and width of seeds produced by *CLE8*-overexpressing plants and *wox8-1* mutant seeds significantly increase and decrease, respectively, compared with those of wild-type seeds. *WOX8* expression significantly increases in CLE8-overexpression lines, indicating that *CLE8* promotes its expression (Fiume and Fletcher, 2012; Song et al., 2013). Some CLE peptides likely interact with hormones. For example, CLE26 expressed at the phloem pole, regulated root architecture, and its expression is significantly enhanced by auxin treatment. CLE26 can affect the activity of the polar auxin transporter in the auxin signaling (Czyzewicz et al., 2015). The shoot growth is related to the root-expressed CLE6 under the gibberellin effect (Bidadi et al., 2014). In addition to development and hormone response, CLE also mediates responses to various abiotic stress. The expression of CLE25 is increased when the root is hydropenic, and then the root-derived CLE25 peptide moves into the leaves to modulate the closure of the stoma (Takahashi et al., 2018). In response to low-sulfate conditions, the expression of CLE2 and CLE3 in roots is reduced, which could inhibit lateral root development (Dong et al., 2019).

*CLE* genes are widely present in plants and even in several plant-parasitic nematodes. Three *CLE*s expressed in the maize endosperm (*Esr1*, *Esr2*, and *Esr3*) are supposed to be involved in signal transduction between embryo and endosperm during early development (Bonello et al., 2000). *ZmCLE7* and *ZmFCP1* are CLV3 homologs, and promoter editing performed by CRISPR-Cas9 increases many grain-yield-related traits in maize (Liu et al., 2021). Two *CLE*s from *Lotus japonicus* (*LjCLE-RS1* and *LjCLE-RS2*) are repressors of excess root nodulation (Okamoto et al., 2013). An *AtCLE19*-like gene with high expression in the flower bud, pistil, and embryo of *Brassica napus* was ectopically expressed in *A. thaliana*, resulting in large heads (Fiers et al., 2004). The mutations of *BnCLV3*s induced by CRISPR/Cas9 could result in multilocular siliques and an increase in seed production (Yang et al., 2018). Similarly, *BrCLV3* mutations conferred multicellular pods in *Brassica rapa* (Fan et al., 2014). In *Raphanus sativus*, overexpression of *RsCLE2* and *RsCLE19* increased the number of xylem elements (Gancheva et al., 2016). Moreover, *CLE*s, which may assist the infection process, are identified in cyst nematodes (Wang J. et al., 2011). Current research suggests that the *CLE*s are highly conserved and involved in biological evolution, especially in plants.

Recent genome-wide analyses have identified CLE genes in many plants (Zhang et al., 2014; Hastwell et al., 2015; Gancheva et al., 2016; Han et al., 2016; Han et al., 2020). The allotetraploid species *B. napus* (2n = 4x = 38, A_n_C_n_), an important oil crop, was formed from the hybridization between *B. rapa* and *Brassica oleracea* (2n = 2x = 18, C_o_) at about 7,500 years ago; therefore, the phylogenetic relationship of *Brassica* provides a good basis for studying the evolution of gene family (Allender and King, 2010). Based on bioinformatics and comparative genomic approaches, this study performed a multidimensional investigation for *CLE*s in rapeseed and its diploid progenitors, including genome-wide identification, molecular characterization, phylogenetic analysis, synteny analysis, expression profiling in different tissues, and regional association mapping analysis. The results will provide useful information for biological functions and molecular evolution in *Brassica*, which also supply candidate genes for genetic improvement in rapeseed breeding.

## Materials and methods

### Identification of *CLE*s in Brassicaceae

Multiple TBLASTN and BLASTN searches were performed for CLEs identification in *B. napus* reference genome (http://brassicadb.org/brad/downloadOverview.php) (Wang et al., 2015) based on *A. thaliana CLE* [expected threshold (e−10)]. The results were then validated by the Conserved Domain Database (CDD) in the National Center for Biotechnology Information (NCBI) (https://www.ncbi.nlm.nih.gov/cdd) (Lu et al., 2020) and Modular Architecture Research Tool SMART (http://smart.embl-heidelberg.de/) (Letunic et al., 2020) to confirm the authenticity of the CLE domain in the open reading frame. Open reading frames of homologous chromosome regions were confirmed for potential unannotated or truncated duplicates of CLEs. *BrCLE*s in *B. rapa* and *BoCLE*s in *B. oleracea* (http://brassicadb.org/brad/downloadOverview.php) were obtained as described above to explore the evolution of *CLE*s in *Brassica*.

### Gene structure, conserved motifs, and *cis*-acting regulatory elements analysis

Gene structures and conserved motifs for the *CLE*s were constructed *via* TBtools. Logo diagrams used to define consensus sequences were obtained using multiple sequence alignments for each BnCLE peptide group (I–V), including *A. thaliana*, *B. rapa*, and *B. oleracea* by TEXshade (Beitz, 2000). The signal peptides of CLEs were identified *via* SMART (Letunic et al., 2020). PlantCARE was carried out to predict the promoters in the 2-kb region before the start codon for the *cis*-element identification (http://bioinformatics.psb.ugent.be/webtools/plantcare/html/) (Magali, 2002). Gene Structure Display Server (GSDS 2.0) (http://bioinformatics.psb.ugent.be/webtools/plantcare/html/) was used to exhibit the gene structure (Hu et al., 2015), and the heatmap was visualized by R package (https://cran.r-project.org/).

### *CLE* gene duplication pattern and synteny analysis

In order to investigate the synteny relationship of CLEs in Brassicaceae, all the *CLE*s were searched as “syntenic genes” in Brassicaceae Database (BRAD) (http://brassicadb.cn/#/). At the same time, TBtools (Chen et al., 2020) was used to detect gene duplication patterns and verify the synteny relationship, as well as calculate the ratio of non-synonymous to synonymous substitutions (Ka/Ks) of syntenic gene pairs. Orthologous *CLE*s located on syntenic chromosome blocks were displayed by Circos software (Krzywinski et al., 2009).

### Phylogenetic analysis of CLEs

Multiple alignments of the CLE peptide sequences from the four Brassicaceae species were performed by the ClustalW (Larkin, 2007). Phylogenetic analysis was generated using the MEGA7 soft with the maximum likelihood (ML) method, 1,000 bootstrap replications, and the JTT+G model (Kumar et al., 2016). The tree was visualized using Evolview (https://www.evolgenius.info/evolview/) (He et al., 2016).

### Prediction of protein–protein interactions

Protein–protein interactions in *A. thaliana* were obtained in the STRING database (https://www.string-db.org/). The analysis and demonstration of CLEs interactions in *B. napus* followed the description based on the previous study (Xie et al., 2022).

### Transcriptome expression pattern of *CLE*s

RNA-seq raw data of siliques, leaves, flowers, and stems for *B. napus*, *B. rapa*, and *B. oleracea* were downloaded from NCBI (ProjectID: PRJNA489323); after being filtered with Trimmomatic (Bolger et al., 2014), the clean data without adapters and low-quality bases were aligned with the reference genome (http://brassicadb.org/brad/downloadOverview.php) using hisat2 (Kim et al., 2015). Based on the mapping results, the gene expressions (fragments per kilobase million (FPKM)) were counted by Stringtie (Pertea et al., 2015), and the heatmaps were drawn by R.

### Quantitative real-time PCR analysis

The *B. napus* ZS11 was used in this study. Tissues like flowers, roots, stems, leaves, immature pods, immature seeds, and apical meristems were collected and extracted using an RNeasy Extraction Kit (Invitrogen, Carlsbad, CA, USA). Quantitative real-time PCR (qRT-PCR) was carried out by referring to a formerly described protocol (Zhao et al., 2021). The expression level was displayed by heatmap in R. Expression patterns of five genes were shown by TBtools-eFP (Chen et al., 2020) (http://yanglab.hzau.edu.cn/BnTIR/eFP).

### Genetic variation of *CLE*s in *Brassica napus* core accessions

To investigate the genetic variation of *CLE* genes in *B. napus*, a panel of 204 rapeseed accessions was selected for this work (Zhao et al., 2022). The single-nucleotide polymorphism (SNP) information of *CLE* genes was retained from the previous study. After annotation with SnpEff (Cingolani et al., 2012), the distribution was analyzed to inspect the position of variations. The agronomic traits for three consecutive years (2014–2016) including plant height, branch number, initial branch height, length, silique number, and silique density of main inflorescence, main inflorescence, silique length, seed number per silique, main inflorescence seed density, thousand seed weight, seed weight per silique, and seed weight of the main inflorescence were surveyed and handled with the best linear unbiased prediction (BLUP). To study the potential impact of *CLE* genes on agronomic traits in *B. napus*, SNPs within 30 kb upstream and downstream of *CLE*s were used for the investigation of its potential impact. Regional association analysis considering population structure and relative kinship was conducted by EMMAX (Kang et al., 2010).

## Result

### Identification of CLEs in *Brassica napus* (Bn), *Brassica rapa* (Br), and *Brassica oleracea* (Bo)

A genome-wide analysis of *CLE* genes in *B. napus* and its diploid progenitors was performed involving multiple BLAST queries and iterative queries, followed by domain validation and removal of false positives (i.e., no CLE domain). Finally, a total of 29 (BrCLEs), 32 (BoCLEs), and 70 (BnCLEs) genes were identified (**Table S2**). The total number of *BrCLE*s and *BoCLE*s in the two diploid progenitors was lower than that of *BnCLE*s in the allotetraploid rapeseed, indicating that CLE gene expansion event has occurred in *B. napus* during polyploidization.

The *CLE*s in the three species of *Brassica* species were renamed according to the AtCLEs in *Arabidopsis* based on the naming conventions for the *B. genus* (Ostergaard and King, 2008), with the last letter “a” indicating the highest homology with *Arabidopsis*, and then “b”, and so on. In *B. napus*, the capital letters A and C following “*Bn*” represented the A_n_ and C_n_ subgenomes, respectively.

### Phylogenetic analysis of *CLE*s in Brassicaceae

**Figure 1:**
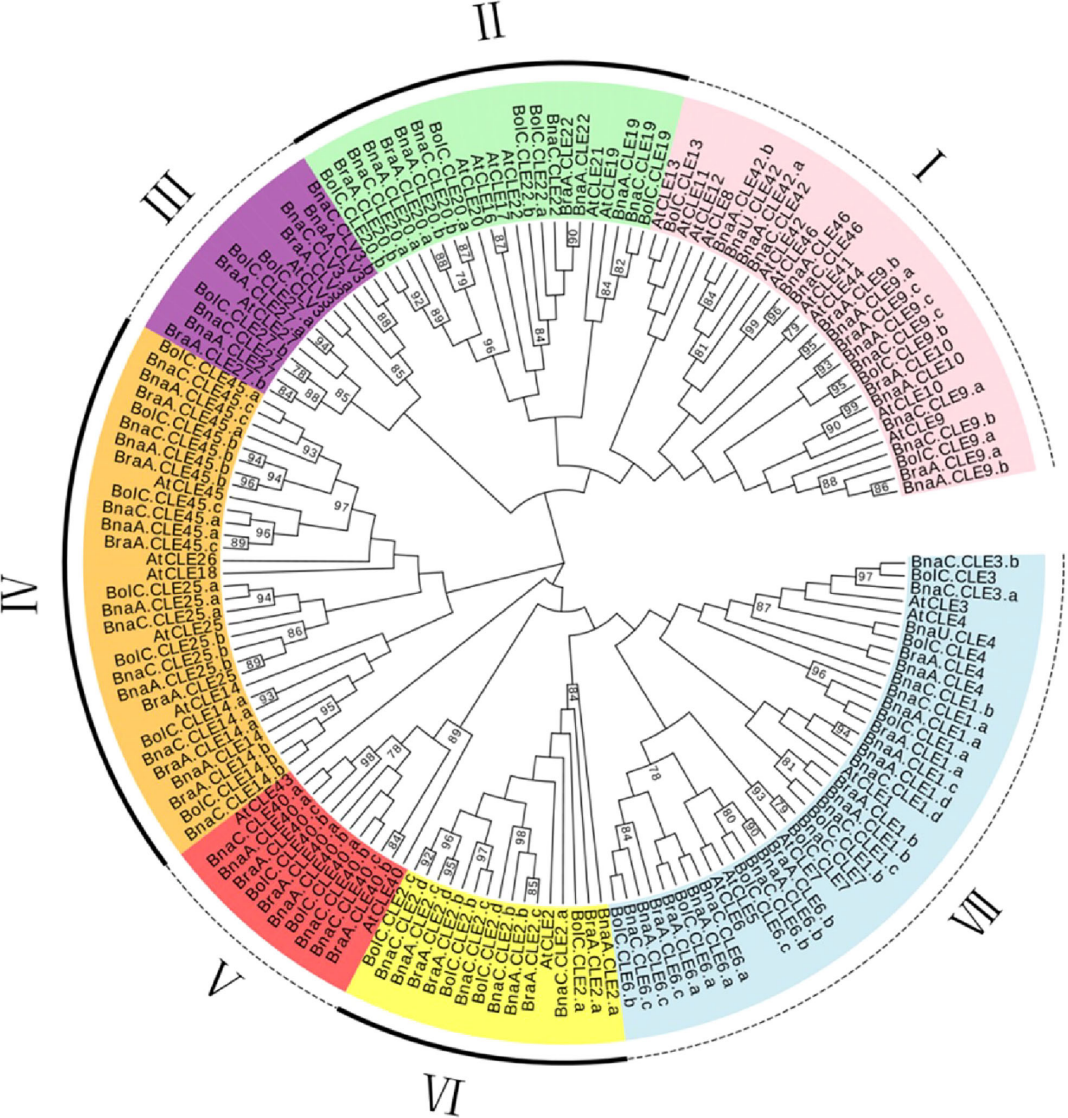
Phylogenetic analysis of *Brassica napus*, *Arabidopsis thaliana*, *Brassica rapa*, and *Brassica oleracea CLE* genes. This unrooted phylogenetic tree was constructed by MEGA 7.0 using the maximum likelihood method with 1,000 bootstrap replicates. Only values above 50% were displayed.

To explore the evolution of the *CLE*s in *Brassica*, we constructed a phylogenetic tree using full-length amino acid sequences from Brassicaceae (**Figure 1**). The results showed *CLE*s clearly divided into seven subfamilies (I to VII). Subfamily V was the largest (42 members), followed by subfamily I (38 members). There were 17, 11, 23, 17, and 14 members in subfamilies II, III, IV, VI, and VII, respectively. The *CLE*s of the four species were distributed in all subfamilies. Most *AtCLE*s in each subfamily matched multiple sets of orthologs from *B. napus* and its two progenitors. A sister pair demonstrated the closest genetic relationship in a phylogenetic tree. A total of 56 sister pairs were observed. The majority of the sister pairs were orthologous gene pairs between the A_n_ (or C_n_) subgenomes of *B. napus* and *B. rapa* (or *B. oleracea*), with 18 A_n_–A_r_ pairs and 19 C_n_–C_o_ pairs. These results supported that the gene duplication events happened in the *B. napus* genome and indicated that *CLE* orthologous genes of distinct subfamilies were highly conserved in the respective genome.

### Duplication pattern and chromosome localization analysis of *CLE*s

**Figure 2:**
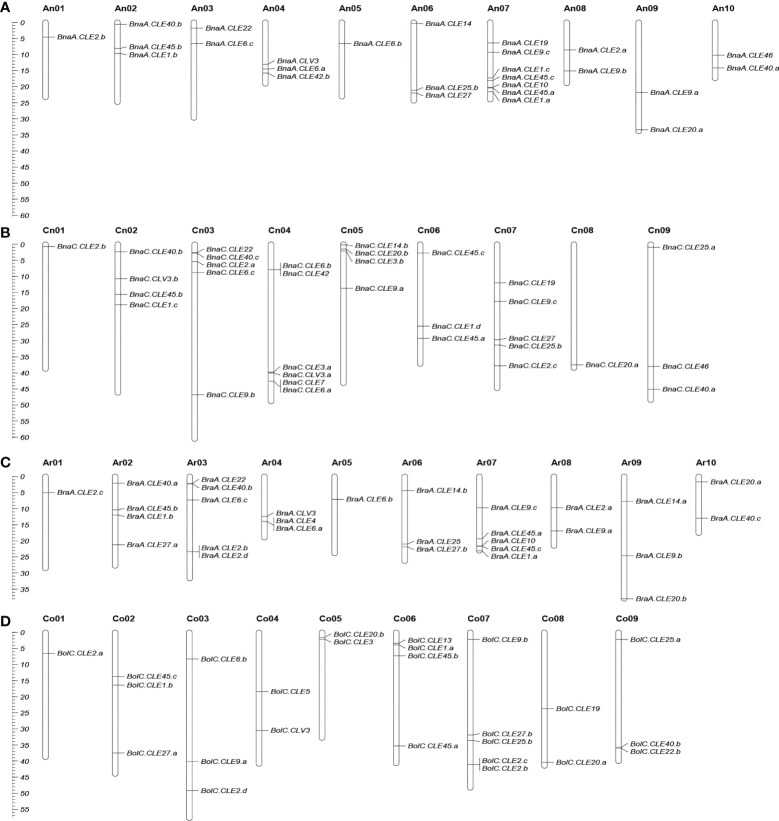
Chromosomal location of *CLE*s in *Brassica napus* **(A, B)**, *Brassica rapa* **(C)**, and *Brassica oleracea* **(D)**. Partial *CLE*s in *B. oleracea* and *B. napus* located in unassembled scaffolds were not shown. The scale on the left is in megabases (Mbs).

The chromosomal locations of *BnCLE*s, *BrCLE*s, and *BoCLE*s were investigated according to their physical positions (**Figure 2**). The *BnCLE*s were asymmetrically distributed on the 19 chromosomes in *B. napus*. There were a total of 32 *BnCLE*s in the A_n_ subgenome and 36 in the C_n_ subgenome, which were similar to those in *B. rapa* (A_r_, 29) and *B. oleracea* (C_o_, 32). The remaining two *BnCLE* genes were located on unanchored scaffolds (**Table S2**). Each chromosome harbored at least one *CLE* gene. Chromosome A_n_07 in *B. napus* carried the most *CLE*s (seven *CLE*s). On chromosomes A_n_01, C_n_01, and C_n_08, only one *CLE* gene was found. Furthermore, many *CLE*s retained their relative position in A_r_ and A_n_, whereas only a portion of *CLE*s retained their relative position in C_o_ and C_n_. For example, the same number of *CLE*s in Ar01–An01, Ar05–An05, Ar06–An06, and Ar08–An08 was observed, which also showed similar locations in the chromosomes (**Figure 2**).

We searched four duplicated types in each Brassicaceae species, including dispersed, proximal, tandem, and whole-genome duplication (WGD) (**Table S3**). We found that 56 of 70 *BnCLE*s were derived from segmental duplication/whole-genome triplication (WGT). Therefore, it appeared that segmental duplication/WGT played an important role in the *BnCLE* expansion. In addition, we examined *CLE* gene expansion patterns in *B. oleracea* and *B. rapa*, finding that most *BoCLE*s (53.1%; 17/32) and *BrCLE*s (89.7%; 26/29) were also derived from segmental duplication/WGT (**Table S3**). One cluster of *CLE* tandem repeat genes was only present in *B. rapa* and *B. oleracea*, but not in *B. napus*.

### Structure analysis of the *CLE*s

**Figure 3:**
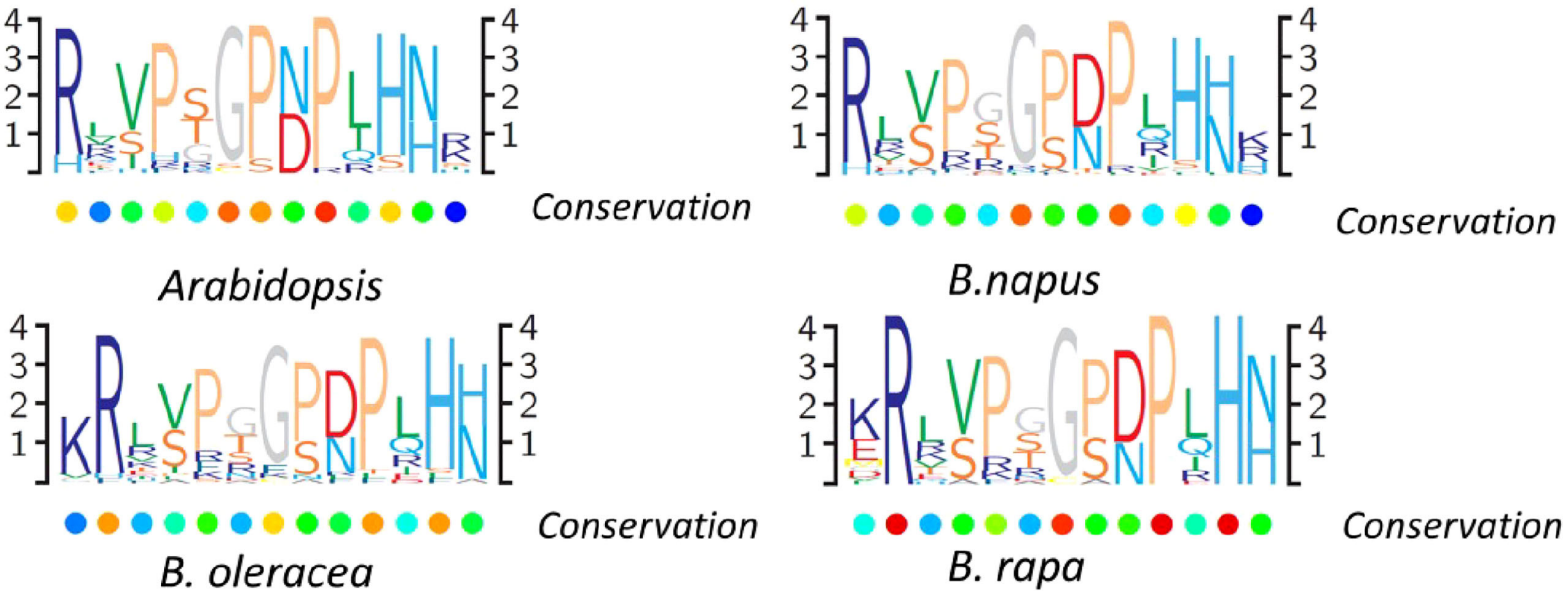
CLAVATA3/EMBRYO SURROUNDING (CLE) domain consensus sequences from *Brassica napus*, *Brassica rapa*, *Brassica oleracea*, and *Arabidopsis* pre-propeptides. Logo diagrams illustrate the 13 amino acid CLE domain consensus sequences, determined from multiple sequence alignments generated for each species.

A great amount of the identified *CLE*s lacked predicted introns, except for 20 genes, including one to three introns (**Table S2**, **Figure S1**). Like other plants, the multiple protein sequence alignment of CLE pre-propeptides in *B. napus* contained a signal peptide at the N-terminus, a central variable domain, and a CLE domain at the C-terminus. The signal peptide sequences and the variable domain among distinct genes were not highly conserved, except between orthologous genes. The highly conserved CLE domain that contained approximately 12/13 aa and residues at different positions had different levels of conservation. To study the sequence of the conserved CLE domains and the degree of their conservation in different Brassicaceae species, multiple sequence alignment was used to generate the protein sequence logos in *B. rapa*, *B. oleracea*, *B. napus*, and *Arabidopsis* (**Figure 3**). The results showed that conserved residues patterns of the CLE domain were remarkably similar in these four plants (**Figure 3**). The conservation of residues 1, 4, 6, 8, 9, 11, and 12 suggested that they may be critical to the function of CLE mature peptides. These results indicated that *CLE* gene family was relatively conserved, while some motif sequences changed slightly during *Brassica* evolution, which possibly contributed to extended special biological function.

**Figure 4:**
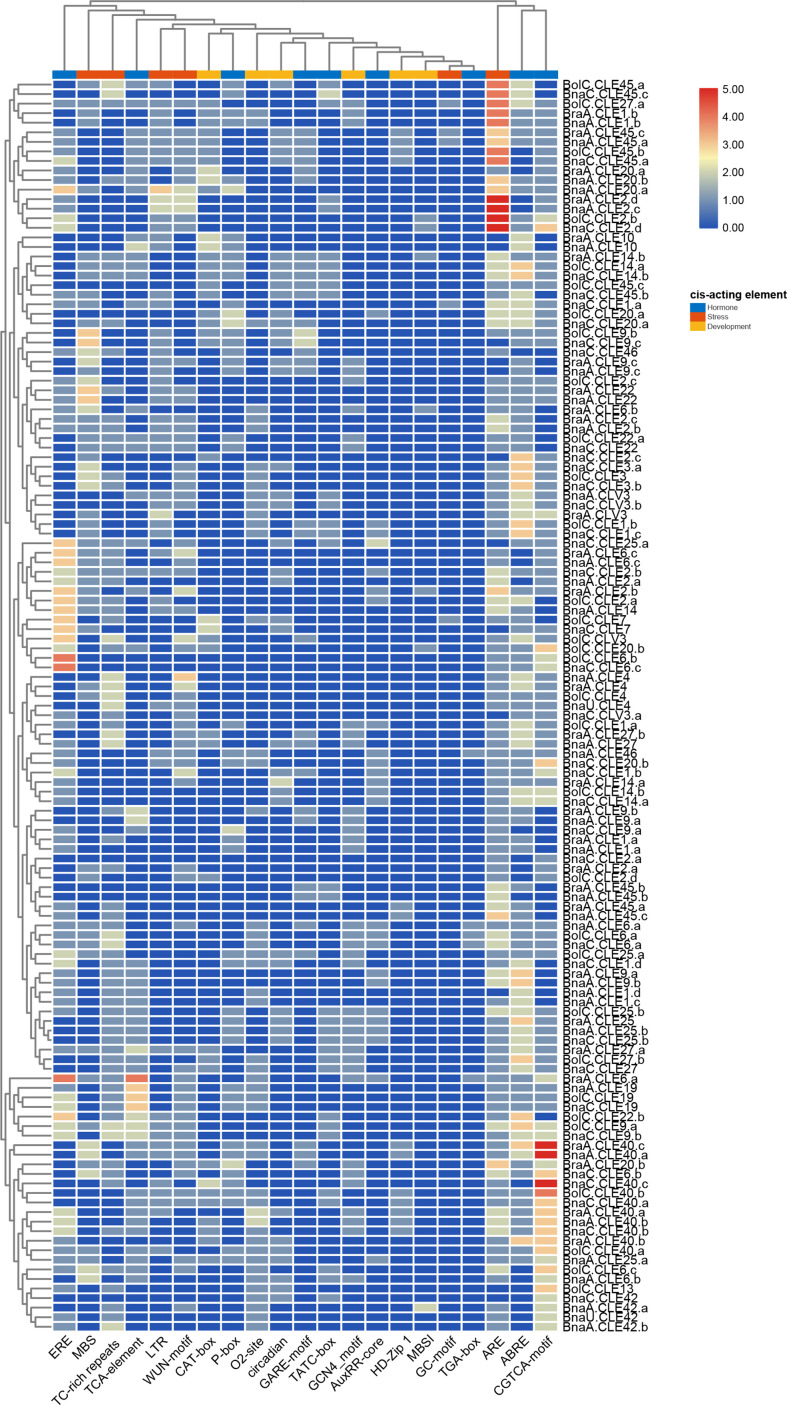
*Cis*-acting elements analysis of *CLE*s in *Brassica napus*, *Brassica rapa*, and *Brassica oleracea*. The color scale bar indicates the numbers of *cis*-acting elements in the promoters.

*Cis*-elements in the promoters can affect gene expression (Davuluri et al., 2003; Klaas, 2009). Therefore, *CLE* gene promoters of these three species were investigated using PlantCARE (Magali, 2002). Four to 12 *cis*-elements involving development, hormone, and stress were identified in each *BnCLE* promoter, while 2–12 and 4–13 were found in *BrCLE*s and *BoCLE*s, respectively (**Figure S2**). ARE has the most elements in these three species and existed in 87.1% (61/70) *BnCLE* promoters, 93.1% (27/29) *BrCLE*s, and 96.9% (31/32) *BoCLE*s; this element is essential for anaerobic induction (**Figure 4**). Hormone-responsive elements involved in abscisic acid responsiveness, methyl jasmonate responsiveness, and ethylene responsiveness were also very common in *CLE* promoters: 80% (56/70), 86.2% (25/29), and 87.5% (28/32) of *BnCLE*, *BrCLE*, and *BoCLE* promoters included ABRE, respectively. In development elements, GCN4 motif involved in endosperm expression (25.7%, 18/70 in *BnCLE*s; 37.9%, 11/29 in *BrCLE*s; and 25%, 8/32 in *BoCLE*s), O2 site involved in zein metabolism regulation (40%, 28/70 in *BnCLE*s; 31%, 9/29 in *BrCLE*s; and 34.4%, 11/32 in *BoCLE*s), CAT-box related to meristem expression (27.1%, 19/70 in *BnCLE*s; 31%, 9/29 in *BrCLE*s; and 37.5%, 12/32 in *BoCLE*s) and circadian (32.9%, 23/70 in *BnCLE*s; 24.1%, 7/29 in *BrCLE*s; and 37.5%, 12/32 in *BoCLE*s) were common. According to the cluster results, some similar *CLE* promoters had similar *cis*-elements, like *CLE4*, *CLE19*, and *CLE42*, while other *CLE*s were clustered into different groups.

### Orthologous relationship and synteny analysis of *CLE* genes

**Figure 5:**
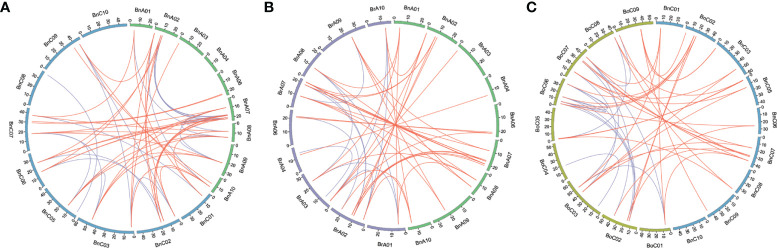
Genome-wide synteny analysis for *CLE*s among *Brassica napus*, *Brassica rapa*, and *Brassica oleracea*. **(A)** Synteny analysis of *CLE*s on An and Cn subgenomes in *B. napus*. **(B)** Synteny analysis of *CLE*s between A_n_ subgenome of *B. napus* and *B. rapa.* **(C)** Synteny analysis of *CLE* genes between Cn subgenome of *B. napus* and *B. oleracea*. Inside the circos, brown lines linked the syntenic orthologs, and blue lines linked the syntenic paralogs.

Syntenic genes are orthologous genes located in syntenic fragments between different species that derive from a shared ancestor. We obtained the syntenic genes of *CLE* genes of *Arabidopsis* in three *Brassica* species by searching “syntenic gene” in BRAD (Wang et al., 2015) and showed these collinearity relationships using Circos (Krzywinski et al., 2009) software between the A_n_ and C_n_ subgenomes of *B. napus* and its two diploid progenitors (**Figure 5**, **Table S4**). A total of 101 *CLE* genes in three *Brassica* species showed conserved synteny with those in *A. thaliana* and were positioned in the same conserved chromosomal blocks, such as A, B, D, E, F, I, J, L, R, U, and Wb (Schranz et al., 2006). In addition, syntenic genes in three *Brassica* species were divided into three fractionated subgenomes, which were specified as LF (least-fractionated), MF1 (medium-fractionated), and MF2 (most-fractionated) according to the extent of gene retention (Chalhoub et al., 2014). There were 41, 31, and 27 *CLE* genes caught in the LF, MF1, and MF2 subgenomes, respectively (**Table S4**). A total of 27 *AtCLE* genes retained corresponding syntenic genes in the three *Brassica* species. The existing forms of syntenic genes in the genomes of three *Brassica* species were different. The first type was that syntenic genes of *AtCLE* were completely preserved in the same block of synteny in the A_r_, C_o_, A_n_, and C_n _subgenomes, such as *AtCLV3*. The second type was that *AtCLE* genes were retained in the A_r_ or/and C_o_ genome but lost in *B. napus* genomes, such as *AtCLE4*, *AtCLE5*, *AtCLE6*, and *AtCLE14*. The third type was that *AtCLE* genes were retained in *B. napus* genome but lost in *B. rapa* or *B. oleracea* genomes, such as *AtCLE1*, *AtCLE9*, and *AtCLE40*. The results showed that the expansion of *CLE* gene family was also accompanied by gene loss.

To comprehend whether natural selection acted on the evolution of *CLE* gene family in *B. napus*, selection pressure analysis was performed on the syntenic *CLE* gene pairs between A_n_ and A_r_, C_n_, and C_o_. The non-synonymous rate (Ka) and synonymous rate (Ks) values were calculated. The Ka/Ks ratio > 1 represents positive selection, the Ka/Ks ratio = 1 represents neutral selection, and the Ka/Ks ratio < 1 represents purifying selection (Nekrutenko, 2002). The Ka/Ks ratios of the syntenic gene pairs are shown in **Table S5**. The Ka/Ks ratios for some *CLE* syntenic gene pairs were >1, such as *BnaA.CLE.1c* and *BnaC.CLE.1d*, *BnaC.CLE.2b* and *BolC.CLE.2b*, *BnaA.CLE.10* and *BraA.CLE.10*, and *BnaA.CLE.27* and *BnaC.CLE.27*, which indicated that these genes were subject to positive selection pressure. Many syntenic gene pairs had no Ka/Ks value in *B. napus* because these two genes had the same sequence or large differences. The rest were less than one, which indicated that they underwent purifying selection during the evolution process and may preferentially perform conserved functions.

### Predicted protein interactions of BnCLEs, BrCLEs, and BoCLEs

**Figure 6:**
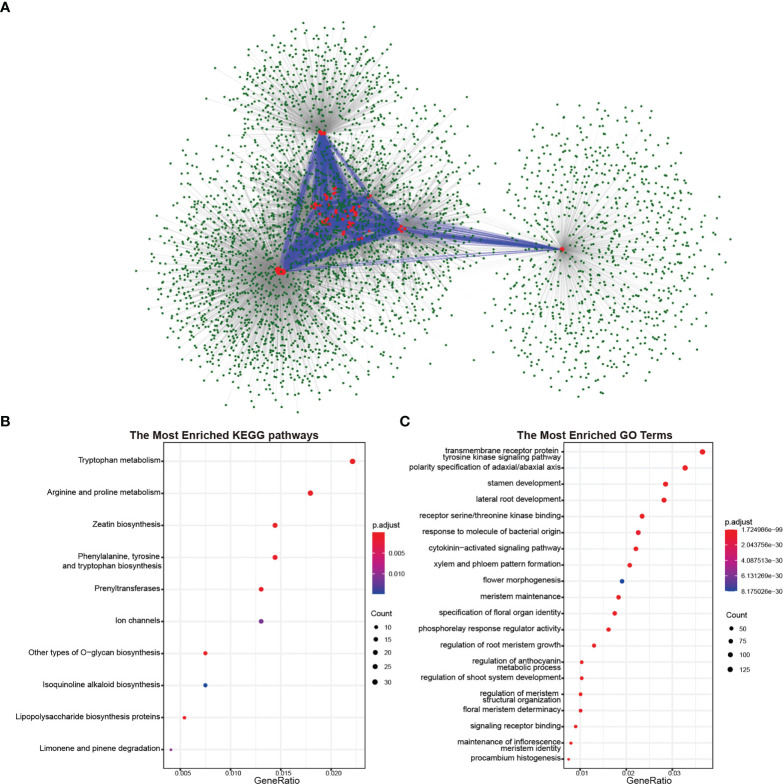
Proteins interacted with CLE proteins in Brassica napus. **(A)** Protein–protein interaction network of CLE proteins in B. napus. The red circles represent the CLE proteins, and the green circles represent proteins interacting with CLE proteins. The blue lines represent the interaction between CLE proteins, and the gray lines represent the interaction between CLE proteins and other proteins. **(B)** Kyoto Encyclopedia of Genes and Genomes (KEGG) pathway enrichment analysis of proteins interacted with CLE proteins. **(C)** Gene Ontology enrichment analysis of proteins interacted with CLE proteins.

As polypeptide hormones, CLEs need to combine with receptor proteins to transmit signals between cells. To investigate the involved biological process in rapeseed and its diploid progenitors, protein–protein interaction networks were predicted based on known protein interactions in *Arabidopsis*. A total of 1,061 proteins *Arabidopsis* proteins interacted with CLEs, resulting in 3,997 proteins in rapeseed (**Figure 6A**). CLEs interacted with other proteins but also interacted with each other. Taking out the interacted genes for Kyoto Encyclopedia of Genes and Genomes (KEGG) enrichment analysis indicated that they played important roles in zeatin biosynthesis, amino acid metabolism (like tryptophan, arginine, and proline), polysaccharide biosynthesis, and ion channels (**Figure 6B**). Gene Ontology (GO) enrichment analysis showed that their molecular functions mainly were receptor serine/threonine kinase binding and phosphorelay response regulator activity; their biological processes were transmembrane receptor protein tyrosine kinase signaling pathway, polarity specification of adaxial/abaxial axis, stamen development, and lateral root development (**Figure 6C**). As to *CLE* genes in *B. rapa* and *B. oleracea*, 1,334 and 1,356 proteins were identified as their interacted proteins, respectively. The Gene Ontology enrichment analysis (**Figures S3**, **S4**) indicated that these proteins were important in the pattern specification process, shoot system morphogenesis, meristem maintenance, root morphogenesis, and leaf development.

### Expression patterns of *CLE* genes in various tissues

According to the RNA-seq data in siliques, leaves, flowers, and stems of *B. napus*, *B. rapa*, and *B. oleracea*, the expression patterns of *CLE*s are illustrated in **Figures S5**-**S7**. The majority of *CLE*s showed low or no expression in all four tissues. Among the expressed *CLE*s, more *CLE* genes were highly expressed in stems in three *Brassica* species, like *BnaC.CLE42*, *BnaA.CLE46*, *BnaC.CLE46*, *BolC.CLE20.b*, *BraA.CLE6.b*, and *BraA.CLE6.c*, whereas *BnaA.CLE9.b*, *BolC.CLE27.a*, *BolC.CLE14.b*, and *BraA.CLE14.a* were highly expressed in siliques. Only a few paralog *CLE* genes had a similar expression pattern, like *BraA.CLE27.a/BraA.CLE27.b*, *BnaC.CLE42/BnaA.CLE42.b/BnaA.CLE42.a/BnaU.CLE42*, *BolC.CLE9.a/BolC.CLE9.b*, and *BolC.CLE22.a/BolC.CLE22.b*. In three *Brassica* species, orthologs like *BnaA.CLE20.b*/*BraA.CLE20.b*/*BolC.CLE20.b* and *BnaC.CLV3.a*/*BraA.CLV3*/*BolC.CLV3* displayed a similar expression pattern. Meanwhile, orthologs in *B. rapa* and *B. oleracea* showed different expression patterns; for example, *BraA.CLE4* was mainly expressed in leaves, whereas *BolC.CLE4* was not. These results suggest that some *CLE* genes have shown functional divergence during their evolution.

**Figure 7:**
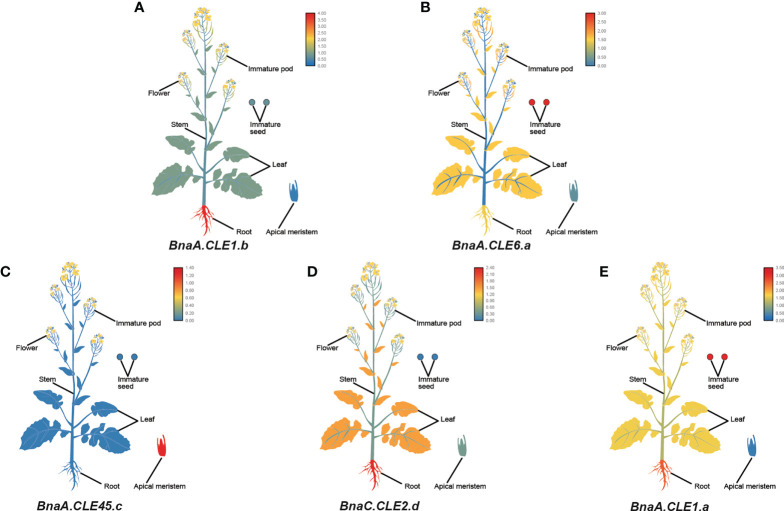
The expression patterns of five selected CLE genes **(A–E)** in Brassica napus plants. Expression data were processed with log10 normalization. The color scale represents relative expression levels from low (blue color) to high (red color).

Furthermore, we performed a qPCR analysis in *B. napus* in various tissues—flowers, roots, stems, leaves, immature pods, immature seeds, and apical meristems. Similar to the transcriptome data, *BnCLE* genes had significantly different expression patterns in different tissues (**Figure S8**). *BnaA.CLE1.b*, *BnaA.CLE2.b*, *BnaC.CLE2.a*/*b*/*c*, *BnaA.CLE14*, and *BnaC.CLE14.b* were more highly expressed in roots than in the other tissues examined (**Figure 7A**), whereas *BnaA.CLE6.a*/*b*, *BnaC.CLE6.b*, *BnaC.CLE7*, *BnaA.CLE4*, *BnaU.CLE4*, *BnaA.CLE1.c*, *BnaC.CLE1.a*/*c*, *BnaA.CLE9.a*/*b*/*c*, and *BnaC.CLE9.a*/*b*/*c* were more highly expressed in immature seeds than in the other tissues (**Figure 7B**). *BnaA.CLE45.c*, *BnaA.CLE19*, *BnaC.CLE19*, and *BnaC.CLV3.a*/*b* were more highly expressed in apical meristems than in the other tissues (**Figure 7C**). *BnaC.CLE2.d* and *BnaC.CLE46* were expressed at high levels in root and stem (**Figure 7D**), while *BnaA.CLE1.a*/*b*, *BnaA.CLE2.a*, *BnaC.CLE6.a*, and *BnaC.CLE14.a* were expressed at high levels in root and immature seeds (**Figure 7E**). The materials and sampling time of the qPCR experiments in this study were different from the data downloaded from NCBI; therefore, the results were not completely consistent. However, there was some similarity: *BnCLE*s showed different expression patterns in different tissues and were modestly expressed in leaves. The expression patterns of most *BnCLE*s pairs were significantly different, suggesting that their roles were dissimilar. For example, *BnaA.CLE1.b* was highly expressed in the roots, whereas *BnaC.CLE1.c* was more highly expressed in immature seeds. *BnaA.CLE1.c* was highly expressed in immature seeds, whereas *BnaA.CLE1.d* was similarly expressed in the different tissues. *BnaA.CLE22* was highly expressed in the stem, whereas *BnaC.CLE22* was more highly expressed in apical meristems. *BnaA.CLE46* was highly expressed in the stem, whereas *BnaC.CLE46* was the more highly expressed root, followed by stem.

### Regional association mapping for *BnCLE*s

A natural population including 204 accessions was selected to analyze the genetic variations of *CLE*s, and a total of 115 SNPs were identified; on average, 1.6 SNPs were detected for each *CLE* gene, which was apparently less than the whole genome level (28.9 SNPs per gene). The total and average number of SNPs in the A subgenome (74 SNPs, 2.2 SNPs/gene) were higher than in the C subgenome (41 SNPs, 1.1 SNPs/gene). Considering *CLE* gene length, the SNP density of the A subgenome was 5.7 SNPs/kb, whereas it was 2.2 SNPs/kb in the C subgenome. Moreover, the SNP distribution among paralogous genes was also unequal; like for BnaA.CLE25.a/BnaA.CLE25.b/BnaC.CLE25.a/BnaC.CLE25.b, the SNP numbers were 9/1/14/0. Finally, SNP annotation implied that 73 SNPs were in exon regions and 37 SNPs could lead to missense mutations.

**Figure 8:**
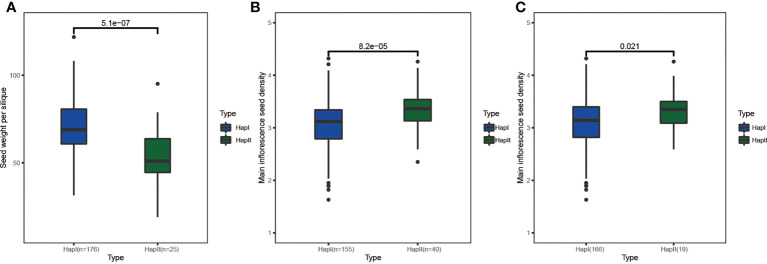
The haplotype analysis of *BnCLE*s for seed weight per silique and main inflorescence seed density. **(A)** *BnaC.CLV3.a*. **(B)** *BnaA.CLE20.b*. **(C)** *BnaC.CLE20.b*.

In this study, 12 agronomic traits (**Figure S9**) and SNPs within the range of 30 kb upstream and downstream of *BnCLE*s were used to study the impact of *BnCLE*s in *B. napus*. A total of 12 *CLE*s were significantly associated with at least one agronomic trait (*p* < 0.0001) (**Table S6**), including plant height, branch height, main inflorescence length, seed weight per silique, silique number, silique density, seed density, and seed weight of the main inflorescence. For example, there was no variation in the gene sequence of *BnaC.CLV3.a*, but SNPs in the upstream region (6 kb) were significantly associated with seed weight per silique; the population was clearly divided into two haplotypes based on the SNPs, and the t-test displayed the significant difference in seed weight per silique between these two groups (**Figure 8A**). According to JASPAR, the SNP region (TCCGTACA) was predicted as the binding site of C2H2 zinc finger factors (SPL3). Moreover, there were another four genes also strongly associated with yield traits, like *BnaA.CLE20.b* and *BnaC.CLE20.b*; SNPs in the upstream region (1.4 and 3.4 kb) were associated with main inflorescence seed density (**Figures 8B, C**). Their interacted proteins were not only enriched in receptor serine/threonine kinase binding (GO:0033612), signaling receptor binding (GO:0005102), and cell–cell signaling involved in cell fate commitment (GO:0045168) but also enriched in GO terms including regulation of meristem structural organization (GO:0009934), maintenance of meristem identity (GO:0010074), and maintenance of root meristem identity (GO:0010078). Therefore, it was speculated that these *CLE*s could affect the development of rapeseed and result in phenotypic variations.

## Discussion

CLE peptides are plant-specific peptide hormones that act as mediators of cell-to-cell communication (Fletcher, 2020). Genome-wide studies on *CLE* gene family have been performed in tomato, soybean, wheat, and populus (Zhang et al., 2014; Hastwell et al., 2015; Han et al., 2016; Li Z. et al., 2019; Han et al., 2020). In this study, systematic identification, classification, evolution, expression, and association mapping analysis were performed in *B. napus*. In total, we identified 70, 32, and 29 *CLE*s in *B. napus*, *B. oleracea*, and *B. rapa*, respectively, which were less than those in the previous work (Han et al., 2020), and the possible reason was more strict parameters used in this work. These *CLE*s were divided into seven subfamilies according to the phylogenetic tree. Furthermore, this work analyzed the relationship of *CLE*s among three *Brassica* species and displayed the similarities and differences of *cis*-acting elements, interacted proteins, and expression patterns. With the aid of a natural population of *B. napus*, the genetic variations of *CLE* genes were uncovered, and several *CLE*s were recognized as candidate genes for important agronomic traits in rapeseed by regional association mapping analysis.

Polyploidization, an important force in the evolution of species (especially magnoliophyte), played an important role in plant adaptation o to new environments (Schemske, 1998; Zhang et al., 2020). Each Brassicaceae genome underwent a WGD event ~35 MYA ago (Bowers, 2003; Yuannian, 2011). Comparative genomic research showed that *Brassica* species experienced triploidy at the genomic level after they diverged from the *Arabidopsis* lineage approximately 20 MYA ago (Chalhoub et al., 2014; Liu et al., 2014). Therefore, it is apparent that the *Brassica* genome underwent paleopolyploidization (Bowers, 2003; Lysak et al., 2005). The differentiation of *B. rapa* and *B. oleracea* occurred approximately 4.6 MYA ago (Liu et al., 2014), and their natural hybridization formed the *B. napus* about 7,500 years ago (Chalhoub et al., 2014). Segmental duplication also leads to increased gene numbers (Flagel and Wendel, 2009). Most plants have undergone polyploidization events and thus retain a large number of duplicated chromosomal blocks at the genome level (Cannon et al., 2004). For example, in *B. napus*, segmental duplication/WGT is the primary force for *WOX* expansion (Li M. et al., 2019). In this study, nearly 80% of *CLE*s were distributed in syntenic blocks, suggesting that segmental duplication/WGT might contribute significantly to *CLE* expansion in *Brassica*. Tandem duplicated genes were defined as an array of at least two homologous genes within 50 kb (Cannon et al., 2004). However, we only found one pair of tandem duplicated genes in *B. rapa* and *B. oleracea*, suggesting that it is not a major factor for *CLE* expansion in *Brassica*.

Not all duplicated genes are retained in plants; gene loss always occurred due to the genomic sequence rearrangement after hybridization or chromosome doubling (Paterson et al., 2004; Ye et al., 2020). Each *AtCLE* should have three syntenic orthologs in *B. rapa* and *B. oleracea* based on the triploidy hypothesis. However, only *AtCLE5*/*6*/*45* had such a pattern, and the other *AtCLE*s had only one or two (less than three syntenic orthologs) in both species (**Table S3**). Hybridization of *B. rapa* and *B. oleracea* should theoretically result in six copies for each homologous *AtCLE* gene in *B. napus*, and the *CLE* number in *B. napus* is equal to the sum of the homologous genes in *B. rapa* and *B. oleracea*. In fact, only *AtCLE9* has six homologous genes in *B. napus*. Generally, most *CLE*s were lost during the formation of *B. napus*. Orthologs of nine *AtCLE*s were not found in three *Brassica* species. *B. napus* lost 38.02% of genes compared with *A. thaliana* at the genome-wide level (27,169 × 6 *vs.* 101,040), while nearly 63.54% of *CLE*s were lost in *B. napus*. The significantly higher average gene loss rate suggests that strong selection occurred for *CLE*s during evolution.

Theoretically, there were three possible means to lose syntenic *BnCLE*s. First, the loss of the *BrCLE*s or *BoCLE*s resulted in the loss of syntenic *BnCLE*s after *Brassica* genome triplication. *AtCLV3* has one syntenic ortholog (*BrCLV3* and *BoCLV3*) in *B. rapa* and *B. oleracea*, so it has two syntenic *BnCLV3*. Second, the loss of the *CLE*s occurred during the allopolyploidization process. *AtCLE2* has three syntenic orthologs in both *B. rapa* and *B. oleracea*, while it has four syntenic orthologs in *B. napus*. Finally, the two processes together caused the loss of syntenic *BnCLE*s. *AtCLE4* has one syntenic ortholog in both *B. rapa* and *B. oleracea*, whereas it has no syntenic ortholog in *B. napus*. These lost *CLE*s were redundant, and genetic change through loss could potentially lead to adaptive diversity.

Statistical analysis showed that 22 out of 29 *CLE* genes (76%) were positioned on the assembled chromosomes in *B. rapa*, whereas 12 out of 25 (48%) maintained their relative position in *B. oleracea* during the formation of *B. napus*. There are two possible reasons for this finding. One possibility is that the C_n_ subgenome has more abundant transposable elements (TEs) than the A_n_ subgenome (Chalhoub et al., 2014). The presence of TEs in the genome can cause the rearrangement of chromosomal sequences, which affects the genomic structure, including deletion, inversion, and translocation. Second, the C_n_ subgenome underwent more active homologous exchanges than the A_n_ subgenome during polyploidization (Chalhoub et al., 2014).

Only a few CLE peptides are studied in *Brassica*; however, the functions of most AtCLE peptides are characterized. Therefore, *Brassica* CLE functions can be predicted through sequence similarity and phylogenetic analysis. Seven distinct CLE subfamilies were defined using *Arabidopsis* and three *Brassica* species. For example, all *CLV3* genes are clustered into subfamily III, while *Brclv3* and *Bnclv3* cause enlarged meristems and lead to extra organs such as multilocular siliques, similar to the phenotype of *Atclv3* (Fletcher et al., 1999; Fan et al., 2014; Yang et al., 2018). This approach could elucidate the function of unknown CLE peptides. However, many genes contain more than one copy in polyploid species (Chalhoub et al., 2014; Li Z. et al., 2019). These duplicated genes are redundant, and variation in promoters could affect their gene expression (Davuluri et al., 2003; Klaas, 2009; Arsovski et al., 2015), For example, *BrCLE45* have three highly similar copies, but *cis*-elements have significant differences, so their expression patterns were different. Distinct expression patterns are a direct sign that duplicated genes may diverge in different directions, indicating the occurrence of non-functionalization, neo-functionalization, or sub-functionalization at the transcriptional level (Gallagher et al., 2016; Cheng et al., 2018). *BnCLE*s appear to be highly expressed in immature seeds, roots, and stems based on their expression levels in previous studies. Moreover, their interacting proteins are enriched in the development of these organs. Therefore, it is concluded that *CLE*s perform important functions in the biological process of these organs. However, the majority of *CLE*s hardly expressed in the tissues analyzed in this study. Therefore, they were either expressed in other developmental stages or conditions, or they were suppressed after duplication. Promoters that regulate the *CLE*s expression may also play a role. Abundant *cis*-elements involved in hormone response, development, and stress were identified, and their existence made the regulation more flexible, which helps plants adapt to complex environments.

Genetic variations in CLEs were investigated in the associated *B. napus* population; the smaller number of SNPs in CLEs indicated that this gene family sequence was very conserved. SNP density was higher in the A subgenome compared with the C subgenome, which is consistent with other published gene families (Zhu et al., 2020; Wahid et al., 2022; Xie et al., 2022). Twelve *CLE*s were found to be significantly associated with important agronomic traits using regional association mapping analysis. Among them, the variations in the upstream region of *BnaC.CLV3.a* (the orthologs of the famous gene *CLV3*) were significantly associated with seed weight per silique. The sequence of variations was predicted as the binding sites of transcription factor SPL3, which can directly bind to the promoters of many genes to affect flower development (Jung et al., 2016). In *Brassica*, *BrCLV3* and *BnCLV3* control multilocular silique traits and increase seed production (Fan et al., 2014; Yang et al., 2018). Although no variation was detected in the genetic region of *BnaC.CLV3.a* in this natural population, the upstream variations that occurred in the binding sites of transcription factor SPL3 may influence the binding ability, which would possibly regulate the expression of *BnaC.CLV3.a* and affect the yield trait. In summary, the association mapping analysis conducted in this study could provide a better way to explore the significance of *CLE*s in phenotypic variation and offer candidate genes for further genetic improvement breeding in *B. napus*.

## Conclusion

A total of 29, 32, and 70 *CLE*s were identified in *B. rapa*, *B. oleracea*, and *B. napus*, respectively, and divided into seven subfamilies in the phylogenetic tree. The conservation of the CLE domain suggested that the *CLE* family is relatively conserved in its biological function. WGT and segmental duplication were the major contributors to the expansion of *CLE*s in *Brassica* species. Transcriptome and qPCR analyses indicated that *BnCLE*s were highly expressed in immature seeds, roots, and stems. Some *CLE* pairs exhibited different expression patterns in the same tissue, indicating duplicated *CLE* differentiation. Moreover, genetic variations and regional association mapping analysis indicated that 12 *CLE* genes were potential genes for regulating important agronomic traits. In summary, this study was helpful in understanding the molecular evolution and biological function of *CLE*s in rapeseed and its diploid progenitors, which would aid future genetic improvement for high-yield breeding in *B. napus*.

## Data availability statement

The datasets presented in this study can be found in online repositories. The names of the repository/repositories and accession number(s) can be found in the article/**Supplementary Material**.

## Author contributions

YX, MX, and CZ designed the research, analyzed the data, and wrote the manuscript. MS analyzed the data. YX and CT reviewed the manuscript. All authors contributed to the article and approved the submitted version.

## Funding

This study was supported by National Natural Science Foundation of China (32070217), Precursor projects of Guizhou province for biological breeding supporting by science and technology in 2022 (Fine identification and evaluation of crop germplasm resources), Subsidy project from NSFC of Guizhou Academy of Agricultural Sciences (No. [2021] 50), Science and technology project of Shandong Education Department to MS (Grant no. J15LE02), China Postdoctoral Science Foundation funded project to MS (Grant no. 2018M632646), the Young Top-notch Talent Cultivation Program of Hubei Province for CT, and the National Natural Science Foundation of China (31770250).

## Acknowledgments

We thank Zhixian Qiao of the Analysis and Testing Center at IHB for the technical support in RNA-seq analysis.

## Conflict of interest

The authors declare that the research was conducted in the absence of any commercial or financial relationships that could be construed as a potential conflict of interest.

## Publisher’s note

All claims expressed in this article are solely those of the authors and do not necessarily represent those of their affiliated organizations, or those of the publisher, the editors and the reviewers. Any product that may be evaluated in this article, or claim that may be made by its manufacturer, is not guaranteed or endorsed by the publisher.

## References

[B1] Allender C. King G. (2010). Origins of the amphiploid species brassica napus l. investigated by chloroplast and nuclear molecular markers. BMC Plant Biol. 10, 54. doi: 10.1186/1471-2229-10-54 20350303 PMC2923528

[B2] Arsovski A. A. Pradinuk J. Guo X. Q. Wang S. Adams K. L. (2015). Evolution of cis-regulatory elements and regulatory networks in duplicated genes of *Arabidopsis* . Plant Physiol. 169 (4), 2982–2991. doi: 10.1104/pp.15.00717 26474639 PMC4677880

[B3] Beitz E. (2000). T(E)Xshade: shading and labeling of multiple sequence alignments using (LTEX)-T-A 2(epsilon). Bioinformatics 16 (2), 135–139. doi: 10.1093/bioinformatics/16.2.135 10842735

[B4] Bidadi H. Matsuoka K. Sage-Ono K. Fukushima J. Pitaksaringkarn W. Asahina M.. (2014). CLE6 expression recovers gibberellin deficiency to promote shoot growth in *Arabidopsis* . Plant J. 78 (2), 241–252. doi: 10.1111/tpj.12475 24528333

[B5] Bolger A. M. Marc L. Bjoern U. (2014). Trimmomatic: a flexible trimmer for illumina sequence data. Bioinformatics 15, 2114–2120. doi: 10.1093/bioinformatics/btu170 PMC4103590 24695404

[B6] Bonello J. Opsahl-Ferstad H. Perez P. Dumas C. Rogowsky P. (2000). Esr genes show different levels of expression in the same region of maize endosperm. Gene 246, 219–227. doi: 10.1016/s0378-1119(00)00088-3 10767543

[B7] Bowers J. E. (2003). Unravelling angiosperm genome evolution by phylogenetic analysis of chromosomal duplication events. Nature 6930, 433-438. doi: 10.1038/nature01521 12660784

[B8] Cannon S. B. Mitra A. Baumgarten A. Young N. D. May G. (2004). The roles of segmental and tandem gene duplication in the evolution of large gene families in *Arabidopsis thaliana* . BMC Plant Biol. 4 (1), 10. doi: 10.1186/1471-2229-4-10 15171794 PMC446195

[B9] Chalhoub B. Denoeud F. Liu S. Parkin I. A. Tang H. Wang X.. (2014). Early allopolyploid evolution in the post-neolithic *Brassica napus* oilseed genome. Science 345 (6199), 950–953. doi: 10.1126/science.1253435 25146293

[B10] Chen C. Chen H. Zhang Y. Thomas H. Frank M. He Y.. (2020). TBtools: An integrative toolkit developed for interactive analyses of big biological data. Mol. Plant 13 (8), 1194–1202. doi: 10.1016/j.molp.2020.06.009 32585190

[B11] Cheng F. Wu J. Cai X. Liang J. Freeling M. Wang X. (2018). Gene retention, fractionation and subgenome differences in polyploid plants. Nat. Plants 4 (5), 258–268. doi: 10.1038/s41477-018-0136-7 29725103

[B12] Cingolani P. Platts A. Wang L. L. Coon M. Nguyen T. Wang L.. (2012). A program for annotating and predicting the effects of single nucleotide polymorphisms, SnpEff: SNPs in the genome of drosophila melanogaster strain w1118; iso-2; iso-3. Fly 6 (2), 80–92. doi: 10.4161/fly.19695 22728672 PMC3679285

[B13] Czyzewicz N. Shi C. L. Vu L. D. Van De Cotte B. Hodgman C. Butenko M. A.. (2015). Modulation of *Arabidopsis* and monocot root architecture by CLAVATA3/EMBRYO SURROUNDING REGION 26 peptide. J. Exp. Bot. 66 (17), 5229–5243. doi: 10.1093/jxb/erv360 26188203 PMC4526925

[B14] Davuluri R. V. Sun H. Palaniswamy S. K. Matthews N. Molina C. Kurtz M.. (2003). AGRIS: Arabidopsis gene regulatory information server, an information resource of *Arabidopsis* cis -regulatory elements and transcription factors. BMC Bioinf. 4 , 25. doi: 10.1186/1471-2105-4-25 PMC166152 12820902

[B15] Dong W. Wang Y. Takahashi H. (2019). CLE-CLAVATA1 signaling pathway modulates lateral root development under sulfur deficiency. Plants (Basel) 8 (4), 103. doi: 10.3390/plants8040103 PMC6524044 31003469

[B16] Fan C. Wu Y. Yang Q. Yang Y. Meng Q. Zhang K.. (2014). A novel single-nucleotide mutation in a CLAVATA3 gene homolog controls a multilocular silique trait in *Brassica rapa* l. Mol. Plant 7 (12), 1788–1792. doi: 10.1093/mp/ssu090 25122699

[B17] Fiers M. Golemiec E. van der Schors R. van der Geest L. Li K. Stiekema W.. (2006). The CLAVATA3/ESR motif of CLAVATA3 is functionally independent from the nonconserved flanking sequences. Plant Physiol. 141 (4), 1284–1292. doi: 10.1104/pp.106.080671 16751438 PMC1533954

[B18] Fiers M. Hause G. Boutilier K. Casamitjana-Martinez E. Weijers D. Offringa R.. (2004). Mis-expression of the CLV3/ESR-like gene CLE19 in *Arabidopsis* leads to a consumption of root meristem. Gene 327 (1), 37–49. doi: 10.1016/j.gene.2003.11.014 14960359

[B19] Fiume E. (2010). Expression analysis of the CLE signaling gene family in arabidopsis thaliana and functional characterization of CLE8 in seed development. Electronic Thesis Dissertations.

[B20] Fiume E. Fletcher J. (2012). Regulation of *Arabidopsis* embryo and endosperm development by the polypeptide signaling molecule CLE8. Plant Cell 24 (3), 1000–1012. doi: 10.1105/tpc.111.094839 22427333 PMC3336133

[B21] Flagel L. E. Wendel J. F. (2009). Gene duplication and evolutionary novelty in plants. New Phytol. 183 (3), 557–564. doi: 10.1111/j.1469-8137.2009.02923.x 19555435

[B22] Fletcher J. C. (2020). Recent advances in arabidopsis CLE peptide signaling. Trends Plant Sci. 25 , 1005-1016. doi: 10.1016/j.tplants.2020.04.014 32402660

[B23] Fletcher J. Brand U. Running M. Simon R. Meyerowitz E. (1999). Signaling of cell fate decisions by CLAVATA3 in *Arabidopsis* shoot meristems. Science 283 (5409), 1911–1914. doi: 10.1126/science.283.5409.1911 10082464

[B24] Gallagher J. Grover C. Hu G. Wendel J. (2016). Insights into the ecology and evolution of polyploid plants through network analysis. Mol. Ecol. 25 (11), 2644–2660. doi: 10.1111/mec.13626 27027619

[B25] Gancheva M. S. Dodueva I. E. Lebedeva M. A. Tvorogova V. E. Tkachenko A. A. Lutova L. A. (2016). Identification, expression, and functional analysis of CLE genes in radish (Raphanus sativus l.) storage root. BMC Plant Biol. 16 Suppl 1, 7. doi: 10.1186/s12870-015-0687-y 26821718 PMC4895270

[B26] Grienenberger E. Fletcher J. C. (2015). Polypeptide signaling molecules in plant development. Curr. Opin. Plant Biol. 23, 8–14. doi: 10.1016/j.pbi.2014.09.013 25449721

[B27] Han S. Khan M. H. U. Yang Y. Zhu K. Li H. Zhu M.. (2020). Identification and comprehensive analysis of the CLV3/ESR-related (CLE) gene family in brassica napus l. Plant Biol. (Stuttg) 22 (4), 709–721. doi: 10.1111/plb.13117 32223006

[B28] Han H. Zhang G. Wu M. Wang G. (2016). Identification and characterization of the populus trichocarpa CLE family. BMC Genomics 17, 174. doi: 10.1186/s12864-016-2504-x 26935217 PMC4776436

[B29] Hastwell A. Gresshoff P. Ferguson B. (2015). Genome-wide annotation and characterization of CLAVATA/ESR (CLE) peptide hormones of soybean (Glycine max) and common bean (Phaseolus vulgaris), and their orthologues of arabidopsis thaliana. J. Exp. Bot. 66 (17), 5271–5287. doi: 10.1093/jxb/erv351 26188205 PMC4526924

[B30] He Z. Zhang H. Gao S. Lercher M. J. Chen W. H. Hu S. (2016). Evolview v2: an online visualization and management tool for customized and annotated phylogenetic trees. Nucleic Acids Res. 44 (W1), W236–W241. doi: 10.1093/nar/gkw370 27131786 PMC4987921

[B31] Hu B. Jin J. Guo A. Zhang H. Luo J. Gao G. (2015). GSDS 2.0: an upgraded gene feature visualization server. Bioinf. (Oxford England) 31 (8), 1296–1297. doi: 10.1093/bioinformatics/btu817 PMC4393523 25504850

[B32] Jung J. H. Lee H. J. Ryu J. Y. Park C. M. (2016). SPL3/4/5 integrate developmental aging and photoperiodic signals into the FT-FD module in arabidopsis flowering. Mol. Plant 9 (12), 1647–1659. doi: 10.1016/j.molp.2016.10.014 27815142

[B33] Kang H. M. Sul J. H. Service S. K. Zaitlen N. A. Kong S. Y. Freimer N. B.. (2010). Variance component model to account for sample structure in genome-wide association studies. Nat. Genet. 42 (4), 348–354. doi: 10.1038/ng.548 20208533 PMC3092069

[B34] Kim D. Langmead B. Salzberg S. L. (2015). HISAT: A fast spliced aligner with low memory requirements. Nat. Methods 12 (4), 357–360. doi: 10.1038/nmeth.3317 25751142 PMC4655817

[B35] Klaas V. (2009). Unraveling transcriptional control in arabidopsis using cis-regulatory elements and coexpression networks. Plant Physiol. 2 , 535–536. doi: 10.1104/pp.109.136028 PMC2689962 19357200

[B36] Krzywinski M. Schein J. Birol I. Connors J. Gascoyne R. Horsman D.. (2009). Circos: an information aesthetic for comparative genomics. Genome Res. 19 (9), 1639–1645. doi: 10.1101/gr.092759.109 19541911 PMC2752132

[B37] Kumar S. Stecher G. Tamura K. (2016). MEGA7: Molecular evolutionary genetics analysis version 7.0 for bigger datasets. Mol. Biol. Evol. 33 (7), 1870–1874. doi: 10.1093/molbev/msw054 27004904 PMC8210823

[B38] Larkin M. (2007). Clustal W and clustal X v. 2.0. Bioinformatics 23 (21), 2947–2948. doi: 10.1093/bioinformatics/btm404 17846036

[B39] Letunic I. Khedkar S. Bork P. (2020). SMART: recent updates, new developments and status in 2020. Nucleic Acids Res. 49, D458–D460. doi: 10.1093/nar/gkaa937 PMC7778883 33104802

[B40] Li Z. Liu D. Xia Y. Li Z. Niu N. Ma S.. (2019). Identification and functional analysis of the CLAVATA3/EMBRYO SURROUNDING REGION (CLE) gene family in wheat. Int. J. Mol. Sci. 20, 4317. doi: 10.3390/ijms20174319 PMC6747155 31484454

[B41] Liu L. Gallagher J. Arevalo E. D. Chen R. Skopelitis T. Wu Q.. (2021). Enhancing grain-yield-related traits by CRISPR-Cas9 promoter editing of maize CLE genes. Nat. Plants 7 (3), 287–294. doi: 10.1038/s41477-021-00858-5 33619356

[B42] Liu S. Liu Y. Yang X. Tong C. Edwards D. Parkin I.. (2014). The brassica oleracea genome reveals the asymmetrical evolution of polyploid genomes. Nat. Commun. 5, 3930. doi: 10.1038/ncomms4930 24852848 PMC4279128

[B43] Li M. Wang R. Liu Z. Wu X. Wang J. (2019). Genome-wide identification and analysis of the WUSCHEL-related homeobox (WOX) gene family in allotetraploid brassica napus reveals changes in WOX genes during polyploidization. BMC Genomics 20 (1), 317. doi: 10.1186/s12864-019-5684-3 31023229 PMC6482515

[B44] Lu S. Wang J. Chitsaz F. Derbyshire M. Geer R. Gonzales N.. (2020). CDD/SPARCLE: the conserved domain database in 2020. Nucleic Acids Res. 48, D265–D268. doi: 10.1093/nar/gkz991 31777944 PMC6943070

[B45] Lysak M. A. Koch M. A. Pecinka A. Schubert I. (2005). Chromosome triplication found across the tribe brassiceae. Genome Res. 15 (4), 516–525. doi: 10.1101/gr.3531105 15781573 PMC1074366

[B46] Magali L. (2002). PlantCARE, a database of plant cis-acting regulatory elements and a portal to tools for *in silico* analysis of promoter sequences. Nucleic Acids Res. 30 (1), 325–327. doi: 10.1093/nar/30.1.325 11752327 PMC99092

[B47] Nekrutenko A. (2002). The K A/K s ratio test for assessing the protein-coding potential of genomic regions: An empirical and simulation study. Genome Res. 12 (1), 198–202. doi: 10.1101/gr.200901 11779845 PMC155263

[B48] Okamoto S. Shinohara H. Mori T. Matsubayashi Y. Kawaguchi M. (2013). Root-derived CLE glycopeptides control nodulation by direct binding to HAR1 receptor kinase. Nat. Commun. 4, 2191. doi: 10.1038/ncomms3191 23934307

[B49] Ostergaard L. King G. J. (2008). Standardized gene nomenclature for the brassica genus. Plant Methods 4 (1), 10–10. doi: 10.1186/1746-4811-4-10 18492252 PMC2408569

[B50] Paterson A. Bowers J. Chapman B. (2004). Ancient polyploidization predating divergence of the cereals, and its consequences for comparative genomics. Proc. Natl. Acad. Sci. U. S. A. 101 (26), 9903–9908. doi: 10.1073/pnas.0307901101 15161969 PMC470771

[B51] Pertea M. Pertea G. M. Antonescu C. M. Chang T. C. Mendell J. T. Salzberg S. L. (2015). StringTie enables improved reconstruction of a transcriptome from RNA-seq reads. Nat. Biotechnol. 33 (3), 290–295. doi: 10.1038/nbt.3122 25690850 PMC4643835

[B52] Rojo E. Sharma V. Kovaleva V. Raikhel N. Fletcher J. (2002). CLV3 is localized to the extracellular space, where it activates the arabidopsis CLAVATA stem cell signaling pathway. Plant Cell 14 (5), 969–977. doi: 10.1105/tpc.002196 12034890 PMC150600

[B53] Schemske R. D. W. (1998). Pathways, mechanisms, and rates of polyploid formation in flowering plants. Annu. Rev. Ecol. Systematics 29, 467–501. doi: 10.1146/annurev.ecolsys.29.1.467

[B54] Schoof H. Lenhard M. Haecker A. Mayer K. F. X. Jürgens G. Laux T. (2000). The stem cell population of arabidopsis shoot meristems is maintained by a regulatory loop between the CLAVATA and WUSCHEL genes. Cell 100 (6), 635–644. doi: 10.1016/S0092-8674(00)80700-X 10761929

[B55] Schranz M. E. Lysak M. A. Mitchell-Olds T. (2006). The ABC's of comparative genomics in the brassicaceae: building blocks of crucifer genomes. Trends Plant ence 11 (11), 535–542. doi: 10.1016/j.tplants.2006.09.002 17029932

[B56] Song X. Guo P. Ren S. Xu T. Liu C. (2013). Antagonistic peptide technology for functional dissection of CLV3/ESR genes in arabidopsis. Plant Physiol. 161 (3), 1076–1085. doi: 10.1104/pp.112.211029 23321419 PMC3585580

[B57] Takahashi F. Suzuki T. Osakabe Y. Betsuyaku S. Kondo Y. Dohmae N.. (2018). A small peptide modulates stomatal control *via* abscisic acid in long-distance signalling. Nature 556 (7700), 235–238. doi: 10.1038/s41586-018-0009-2 29618812

[B58] Tatsuhiko K. (2006). A plant peptide encoded by CLV3 identified by *in situ* MALDI-TOF MS analysis. Sci. (New York N.Y.) 5788 , 845–848. doi: 10.1126/science.1128439 16902141

[B59] Wahid S. Xie M. Sarfraz S. Liu J. Zhao C. Bai Z.. (2022). Genome-wide identification and analysis of Ariadne gene family reveal its genetic effects on agronomic traits of brassica napus. Int. J. Mol. Sci. 23, 6265. doi: 10.3390/ijms23116265 35682945 PMC9181464

[B60] Wang J. Replogle A. Hussey R. Baum T. (2011). Identification of potential host plant mimics of CLAVATA3ESR (CLE)-like peptides from the plant-parasitic nematode heterodera schachtii. Mol. Plant Pathol. 12 (2), 177–186. doi: 10.1111/j.1364-3703.2010.00660.x 21199567 PMC6640238

[B61] Wang X. Wu J. Liang J. Cheng F. Wang X. (2015). Brassica database (BRAD) version 2.0: Integrating and mining brassicaceae species genomic resources. Database J. Biol. Database Curation 2015, bav093. doi: 10.1093/database/bav093 PMC4653866 26589635

[B62] Xie M. Zuo R. Bai Z. Yang L. Zhao C. Gao F.. (2022). Genome-wide characterization of Serine/Arginine-rich gene family and its genetic effects on agronomic traits of brassica napus. Front. Plant Sci. 13. doi: 10.3389/fpls.2022.829668 PMC8889041 35251101

[B63] Yang Y. Zhu K. Li H. Han S. Meng Q. Khan S.. (2018). Precise editing of CLAVATA genes in brassica napus l. regulates multilocular silique development. Plant Biotechnol. J. 16 (7), 1322–1335. doi: 10.1111/pbi.12872 29250878 PMC5999189

[B64] Ye C. Wu D. Mao L. Jia L. Qiu J. Lao S.. (2020). The genomes of the allohexaploid echinochloa crus-galli and its progenitors provide insights into polyploidization-driven adaptation. Mol. Plant 13 (9), 1298–1310. doi: 10.1016/j.molp.2020.07.001 32622997

[B65] Yuannian J. (2011). Ancestral polyploidy in seed plants and angiosperms. Nature 7345, 97-100. doi: 10.1038/nature09916 21478875

[B66] Zhang L. Wu S. Chang X. Wang X. Zhao Y. Xia Y.. (2020). The ancient wave of polyploidization events in flowering plants and their facilitated adaptation to environmental stress. Plant Cell Environ. 43 (12), 2847–2856. doi: 10.1111/pce.13898 33001478

[B67] Zhang Y. Yang S. Song Y. Wang J. (2014). Genome-wide characterization, expression and functional analysis of CLV3/ESR gene family in tomato. BMC Genomics 15 (1), 827. doi: 10.1186/1471-2164-15-827 25266499 PMC4195864

[B68] Zhao C. Safdar L. B. Xie M. Shi M. Dong Z. Yang L.. (2021). Mutation of the PHYTOENE DESATURASE 3 gene causes yellowish-white petals in *Brassica napus* . Crop J. 9, 1124–1134. doi: 10.1016/j.cj.2020.10.012

[B69] Zhao C. Xie M. Liang L. Yang L. Han H. Qin X.. (2022). Genome-wide association analysis combined with quantitative trait loci mapping and dynamic transcriptome unveil the genetic control of seed oil content in brassica napus l. Front. Plant Sci. 13. doi: 10.3389/fpls.2022.929197 PMC9283957 35845656

[B70] Zhu W. Guo Y. Chen Y. Wu D. Jiang L. (2020). Genome-wide identification, phylogenetic and expression pattern analysis of GATA family genes in brassica napus. BMC Plant Biol. 20 (1), 543. doi: 10.1186/s12870-020-02752-2 33276730 PMC7716463

