# Supplementary figures and images for: Genome-wide identification and comparative analysis of CLE family in rapeseed and its diploid progenitors
Source: Front Plant Sci. 2022 Oct 20;13:998082. doi: 10.3389/fpls.2022.998082 (PMC9632860; doi:10.3389/fpls.2022.998082)

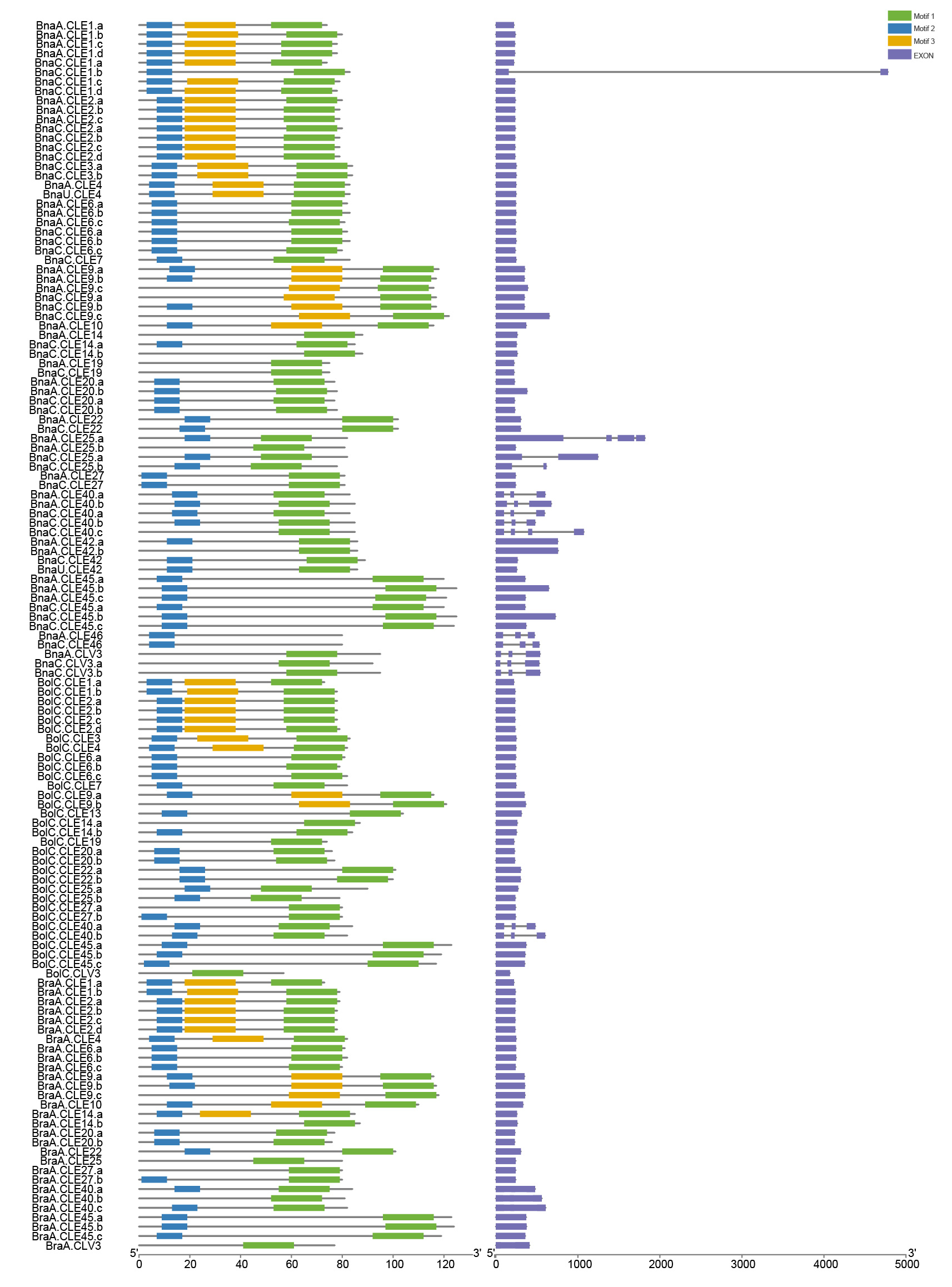

Supplement: Supplementary file 7 [file Image_1.jpeg]

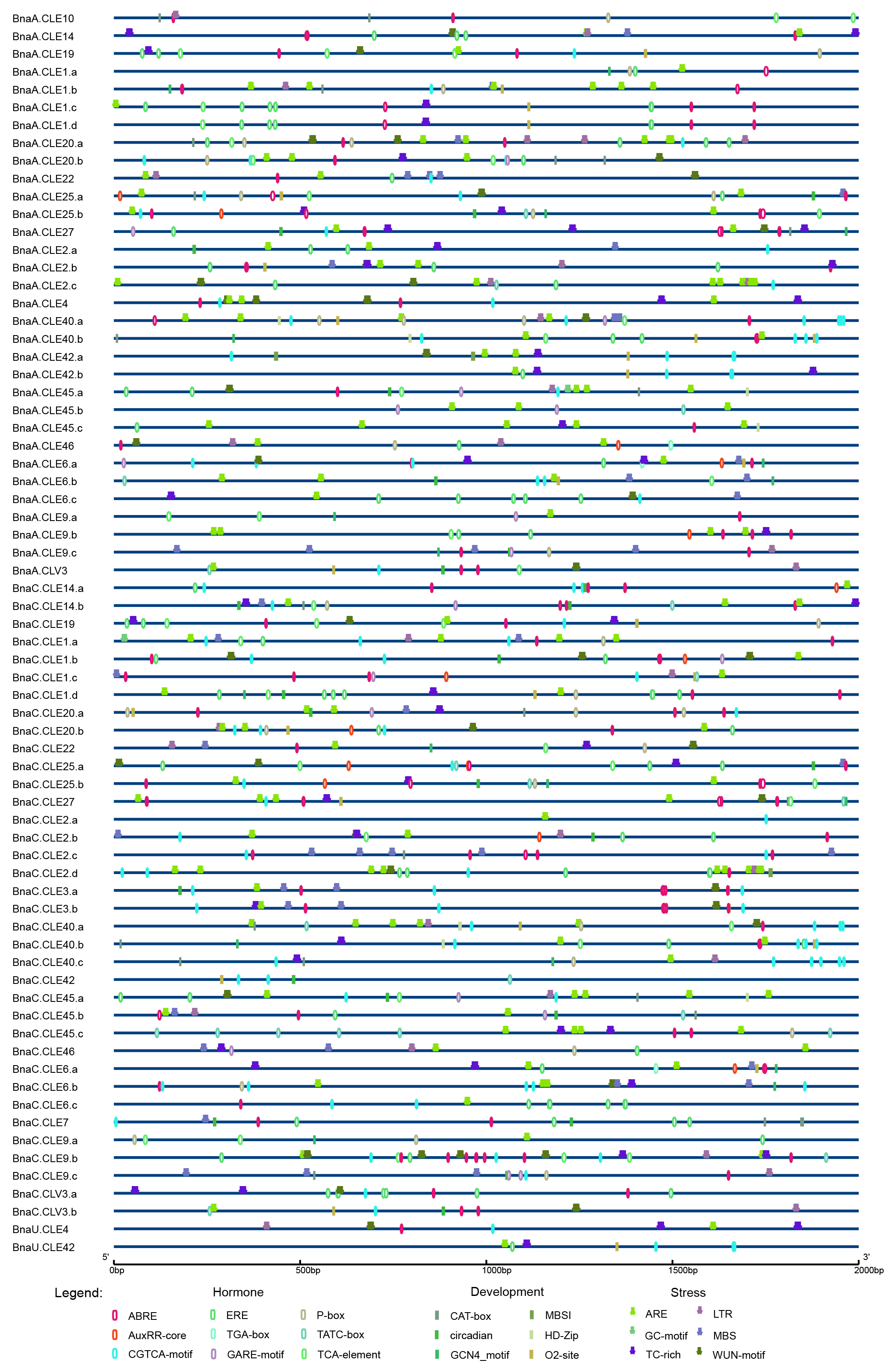

Supplement: Supplementary file 8 [file Image_2.jpeg]

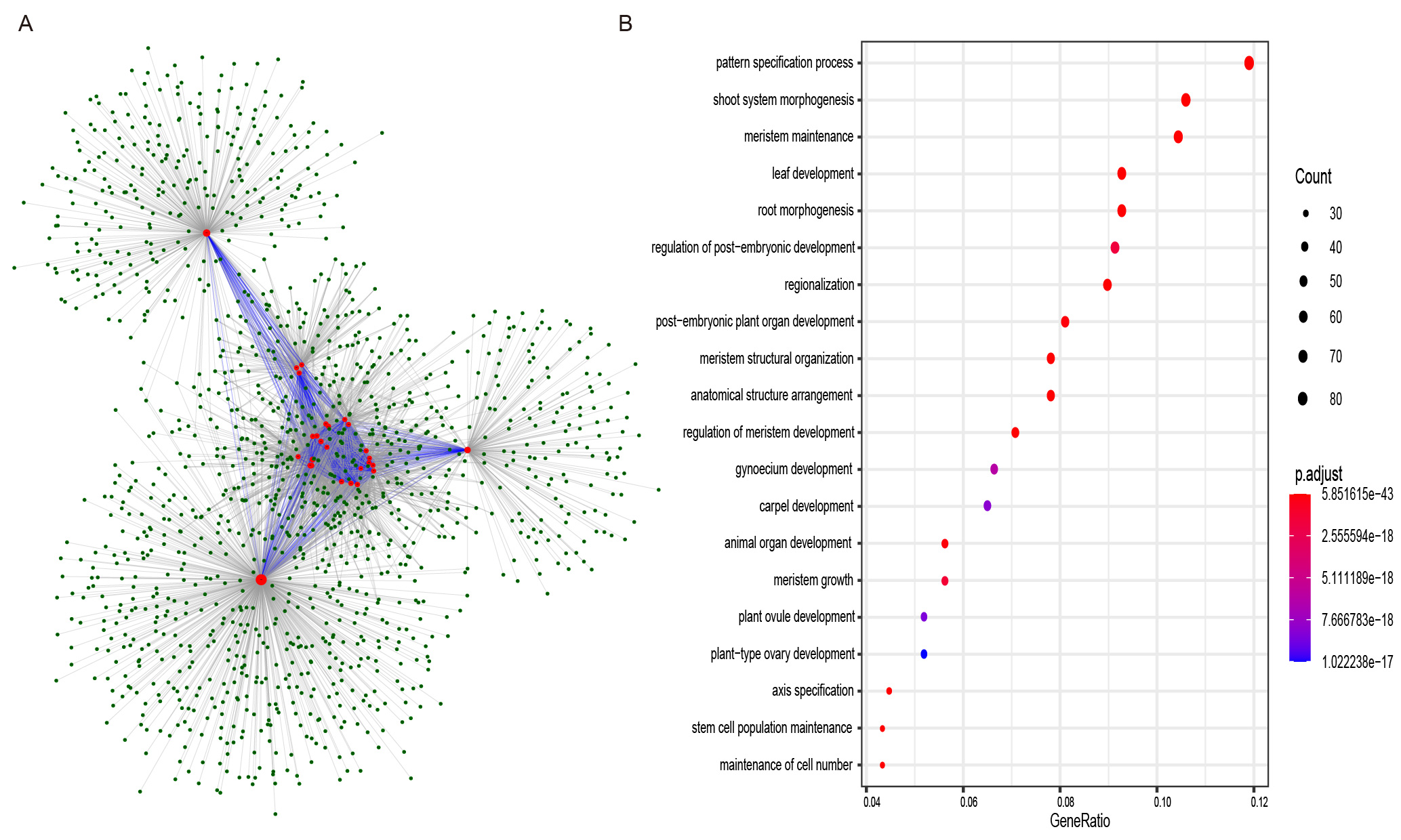

Supplement: Supplementary file 9 [file Image_3.jpeg]

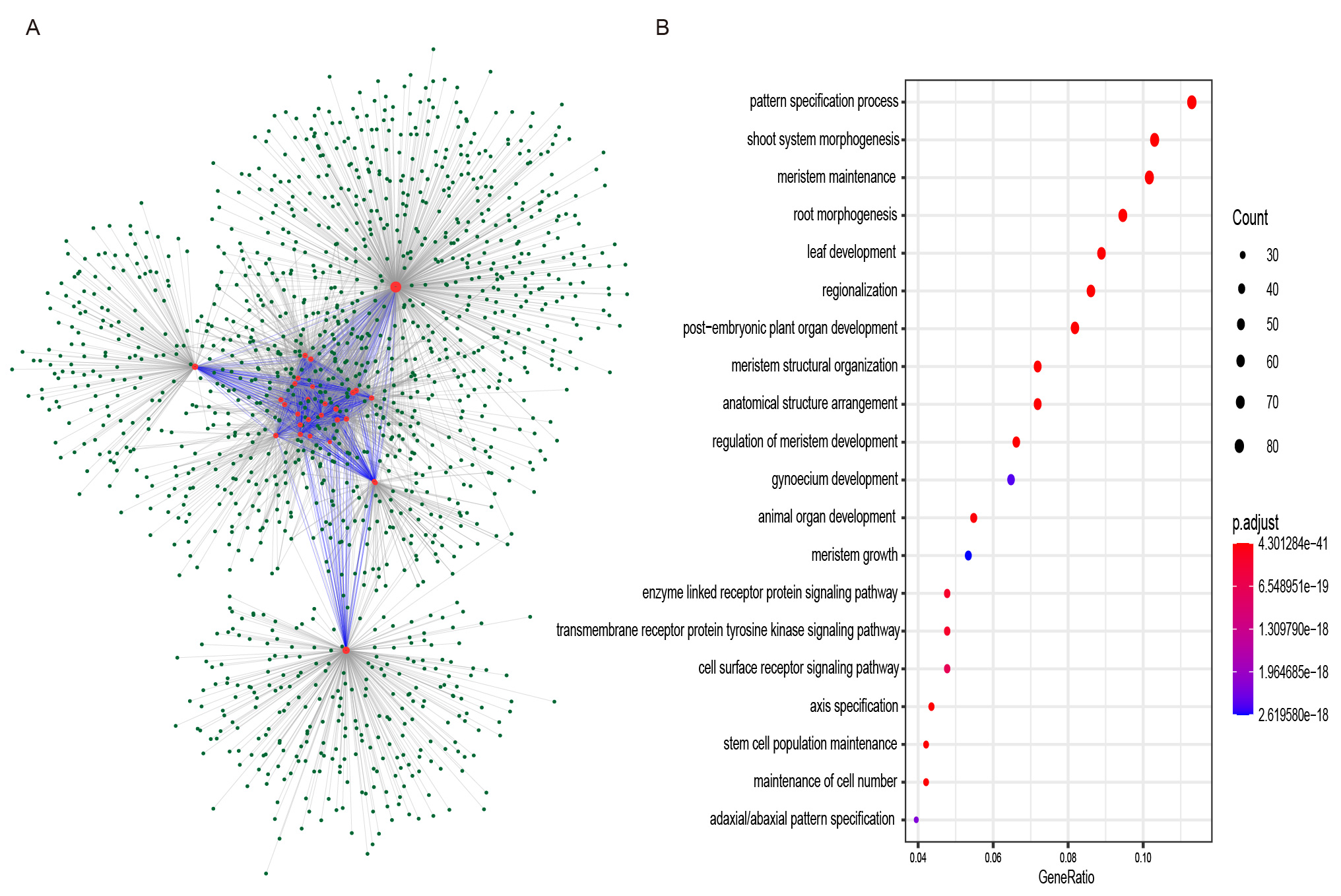

Supplement: Supplementary file 10 [file Image_4.jpeg]

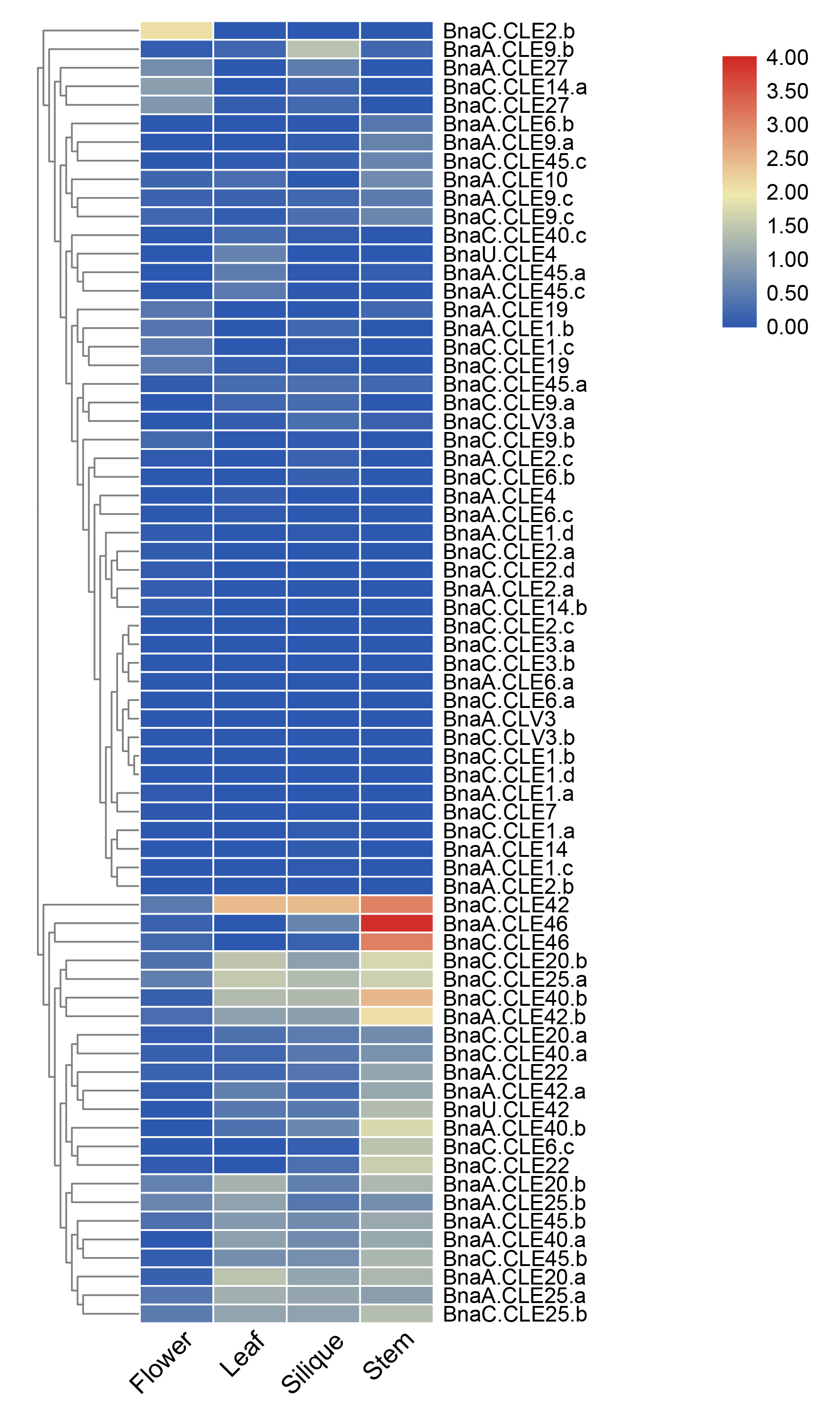

Supplement: Supplementary file 11 [file Image_5.jpeg]

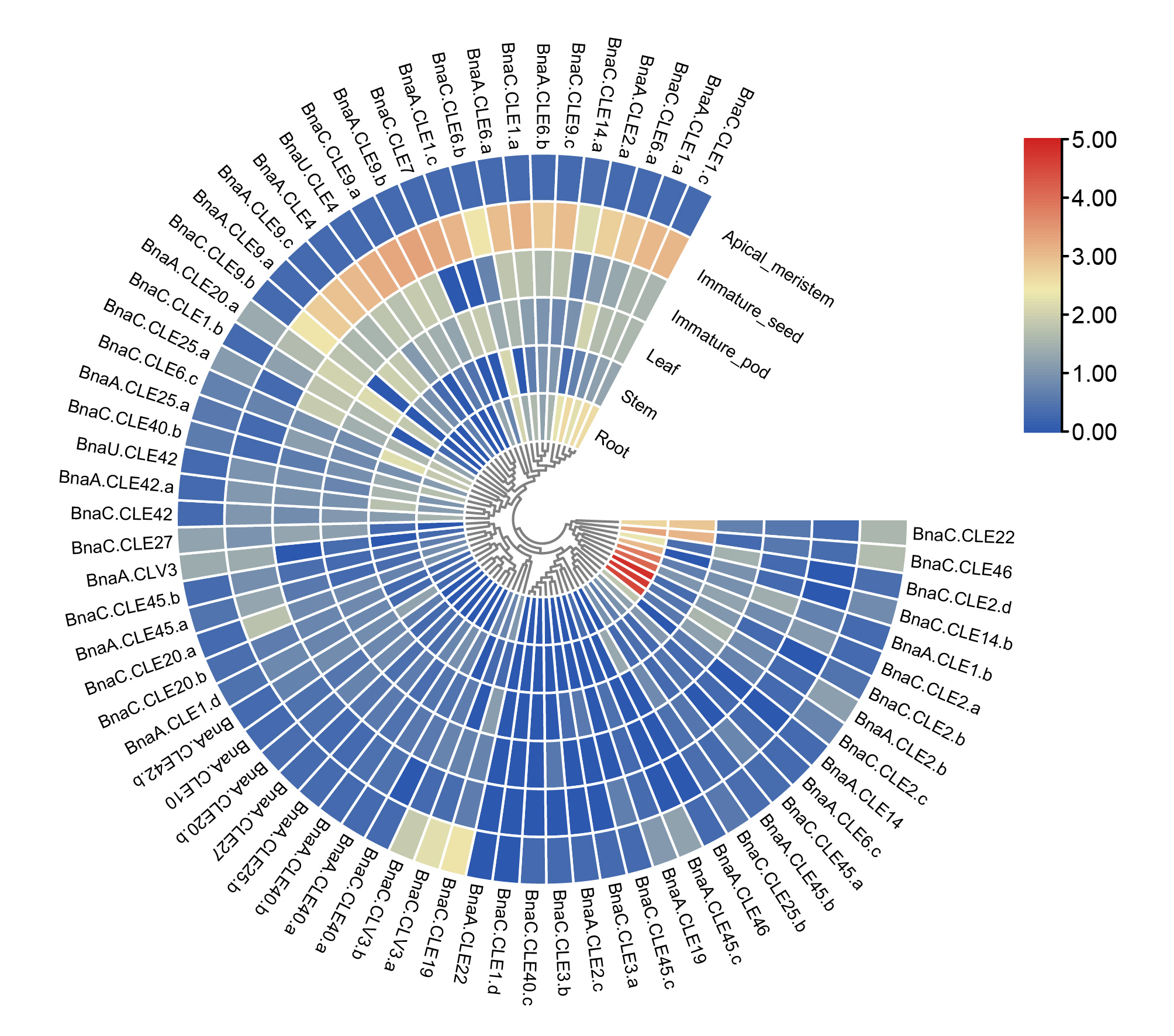

Supplement: Supplementary file 12 [file Image_6.jpeg]

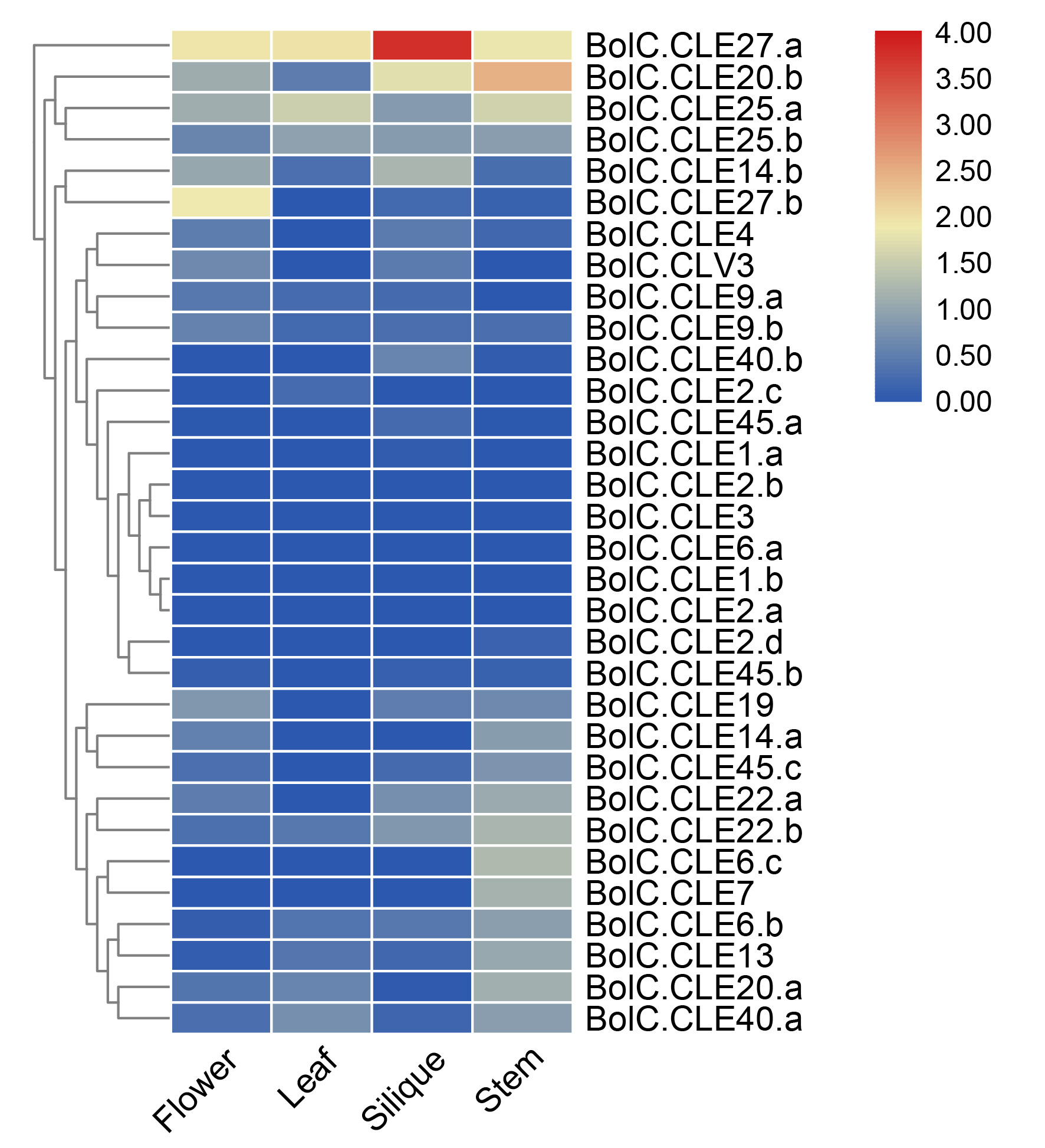

Supplement: Supplementary file 13 [file Image_7.jpeg]

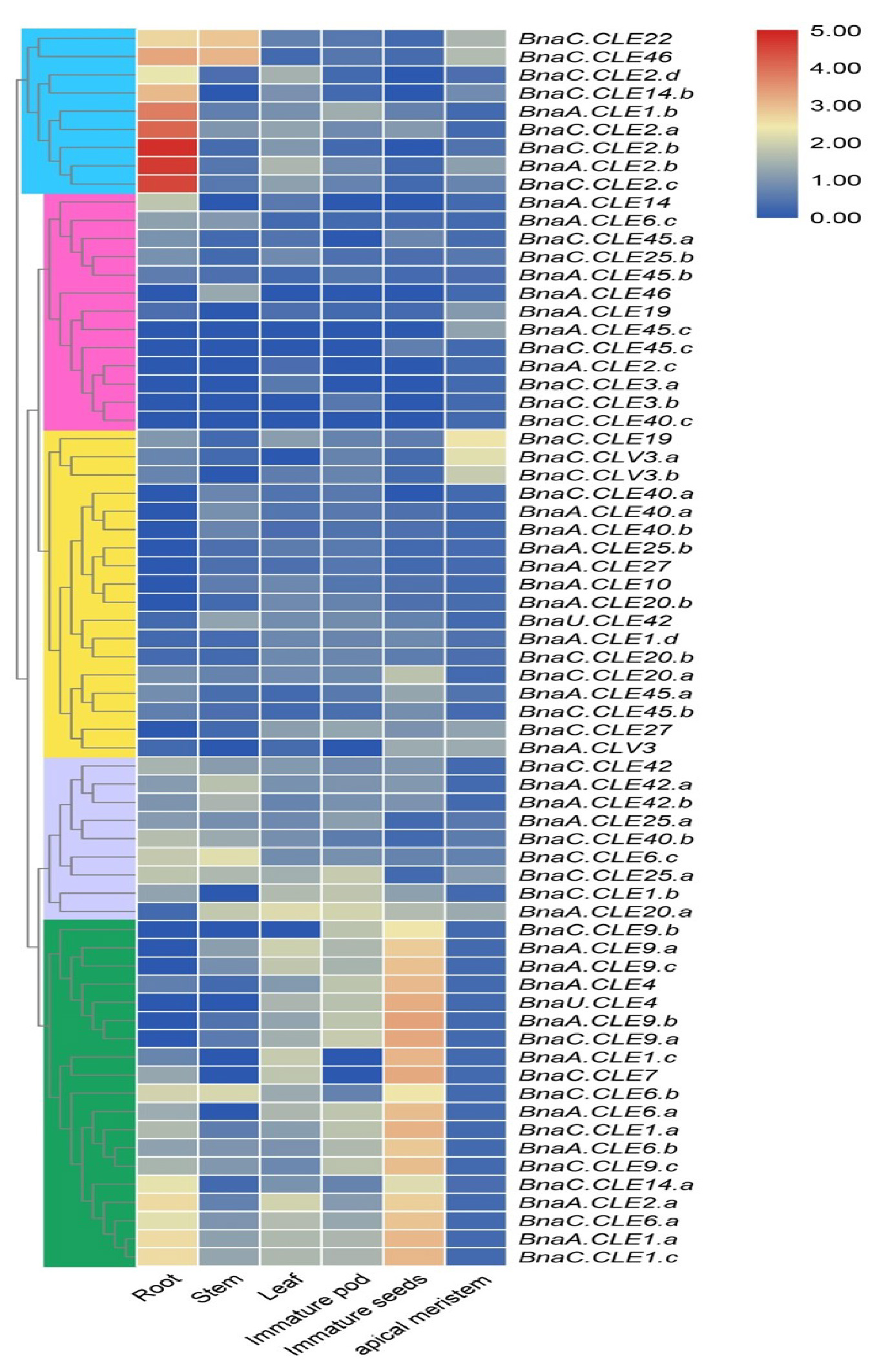

Supplement: Supplementary file 14 [file Image_8.jpeg]

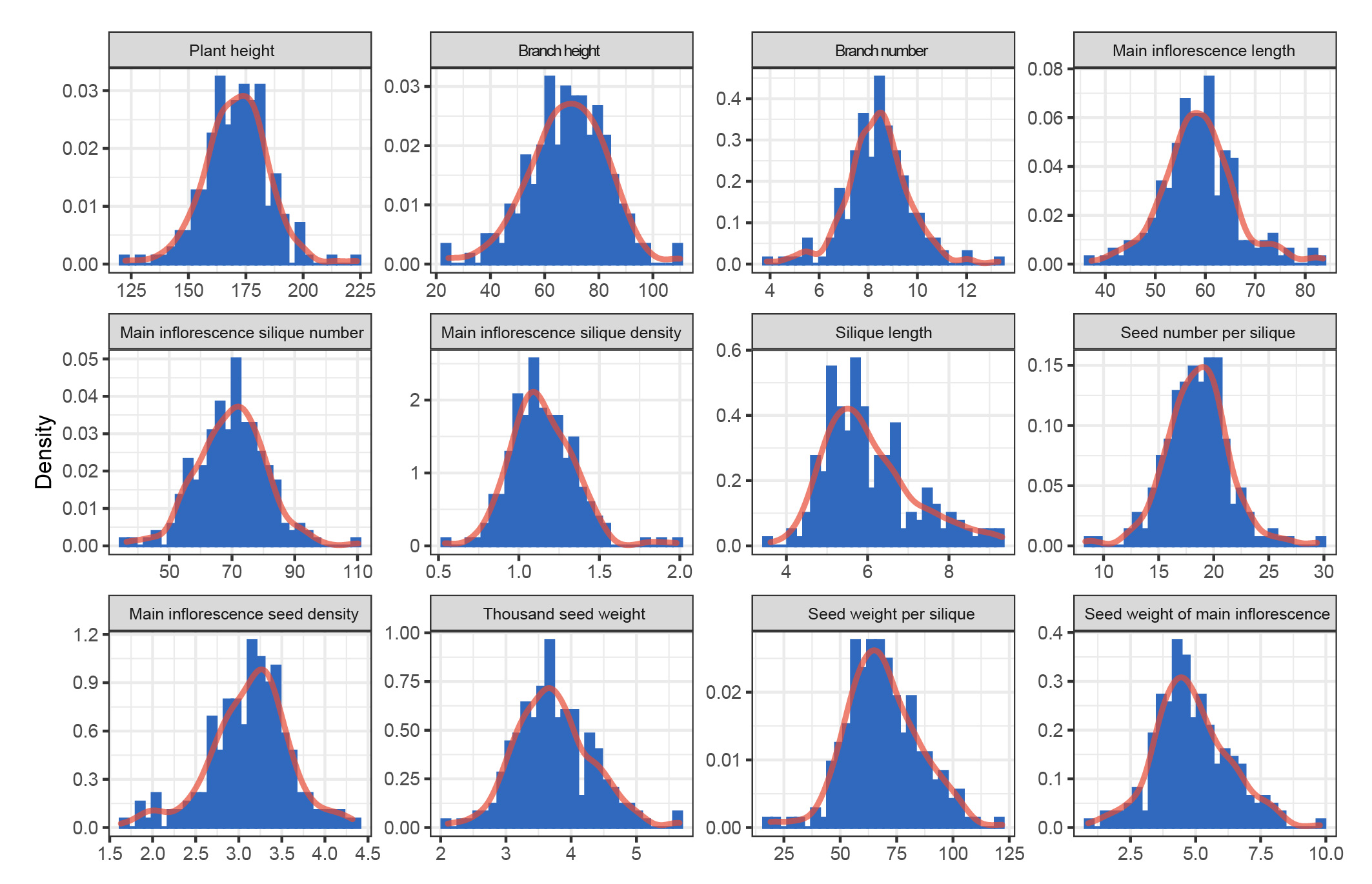

Supplement: Supplementary file 15 [file Image_9.jpeg]
